# Endogenous signalling pathways and caged IP_3_ evoke Ca^2+^ puffs at the same abundant immobile intracellular sites

**DOI:** 10.1242/jcs.208520

**Published:** 2017-11-01

**Authors:** Michael V. Keebler, Colin W. Taylor

**Affiliations:** Department of Pharmacology, University of Cambridge, Tennis Court Road, Cambridge CB2 1PD, UK

**Keywords:** Ca^2+^ puff, Endoplasmic reticulum, IP_3_, IP_3_ receptor, Muscarinic receptor, Phospholipase C

## Abstract

The building blocks of intracellular Ca^2+^ signals evoked by inositol 1,4,5-trisphosphate receptors (IP_3_Rs) are Ca^2+^ puffs, transient focal increases in Ca^2+^ concentration that reflect the opening of small clusters of IP_3_Rs. We use total internal reflection fluorescence microscopy and automated analyses to detect Ca^2+^ puffs evoked by photolysis of caged IP_3_ or activation of endogenous muscarinic receptors with carbachol in human embryonic kidney 293 cells. Ca^2+^ puffs evoked by carbachol initiated at an estimated 65±7 sites/cell, and the sites remained immobile for many minutes. Photolysis of caged IP_3_ evoked Ca^2+^ puffs at a similar number of sites (100±35). Increasing the carbachol concentration increased the frequency of Ca^2+^ puffs without unmasking additional Ca^2+^ release sites. By measuring responses to sequential stimulation with carbachol or photolysed caged IP_3_, we established that the two stimuli evoked Ca^2+^ puffs at the same sites. We conclude that IP_3_-evoked Ca^2+^ puffs initiate at numerous immobile sites and the sites become more likely to fire as the IP_3_ concentration increases; there is no evidence that endogenous signalling pathways selectively deliver IP_3_ to specific sites.

## Abstract (precis)

**Summary:** Ca^2+^ puffs are the building blocks for IP_3_-evoked Ca^2+^ signals. Ca^2+^ puffs evoked by caged IP_3_ or via endogenous signalling pathways initiate at the same fixed intracellular sites.

## INTRODUCTION

Most Ca^2+^ signals in electrically inexcitable cells are initiated by receptors that stimulate phospholipase C (PLC) and, thereby, formation of inositol 1,4,5-trisphosphate (IP_3_). IP_3_ then binds to IP_3_ receptors (IP_3_Rs) to stimulate Ca^2+^ release from the endoplasmic reticulum (ER). Vertebrates express three subtypes of IP_3_R and numerous splice variants but each contributes to formation of tetrameric Ca^2+^-permeable channels that open when they bind both IP_3_ and Ca^2+^ (Foskett et al., 2007; Prole and Taylor, 2016). Higher concentrations of cytosolic free Ca^2+^ concentration ([Ca^2+^]_c_) than those required for IP_3_R activation inhibit IP_3_Rs. Within ER membranes, IP_3_Rs form small clusters (Chalmers et al., 2006; Geyer et al., 2015; Rahman et al., 2009), wherein Ca^2+^ released by an active IP_3_R can stimulate the opening of neighbouring IP_3_Rs that have bound IP_3_ (Alzayady et al., 2016; Taylor and Konieczny, 2016). Hence, both the distribution of IP_3_Rs and the prevailing IP_3_ concentration set the gain on this regenerative Ca^2+^-induced Ca^2+^ release mechanism.

Studies conducted in Ian Parker's laboratory have shown that low concentrations of IP_3_ evoke tiny, local cytosolic Ca^2+^ signals (‘Ca^2+^ blips’) that last just a few milliseconds and reflect the opening of single IP_3_Rs. Higher concentrations of IP_3_ evoke longer-lasting local Ca^2+^ signals (‘Ca^2+^ puffs’) that are typically 5−10 times larger than blips and result from the almost simultaneous openings of a few IP_3_Rs (Parker et al., 1996). Ca^2+^ blips and puffs are the building blocks of all IP_3_-evoked Ca^2+^ signals (Bootman et al., 1997a). As the global [Ca^2+^]_c_ or concentration of IP_3_ increases further, Ca^2+^ puffs become more frequent until they typically trigger a Ca^2+^ wave that spreads regeneratively across the cell (Marchant et al., 1999), at frequencies that increase with the concentration of IP_3_ (Thurley et al., 2014).

Ca^2+^ puffs evoked by either caged IP_3_ (ci-IP_3_) or by IP_3_ produced after activation of endogenous signalling pathways have been observed in numerous cell types. A common theme is the suggestion that Ca^2+^ puffs recur at the same small number of subcellular locations and that these sites often initiate global cytosolic Ca^2+^ signals (Bootman et al., 1997a; Kasai et al., 1993; Marchant and Parker, 2001; Nakamura et al., 2012; Olson et al., 2012; Rooney et al., 1990; Simpson et al., 1997). However, most quantitative analyses of Ca^2+^ puffs that have used total internal reflection fluorescence microscopy (TIRFM) have also been restricted to a single cell type (SH-SY5Y neuroblastoma cells) responding to photolysis of ci-IP_3_ (e.g. Smith and Parker, 2009). There is, therefore, a need to both examine other cells and to compare responses to ci-IP_3_ with those evoked by endogenous signalling pathways. The latter is important because there is evidence that different stimuli that activate endogenous pathways and direct delivery of IP_3_ may each ignite Ca^2+^ puffs at the same small number of Ca^2+^ release sites (Rooney et al., 1990; Thomas et al., 2000), whereas other studies suggest a selective delivery of IP_3_ to specific IP_3_Rs by endogenous signalling pathways (Delmas et al., 2002; Olson et al., 2012; Short et al., 2000; Tovey and Taylor, 2013; Tu et al., 1998; Zhao et al., 1990).

**Fig. 1.**
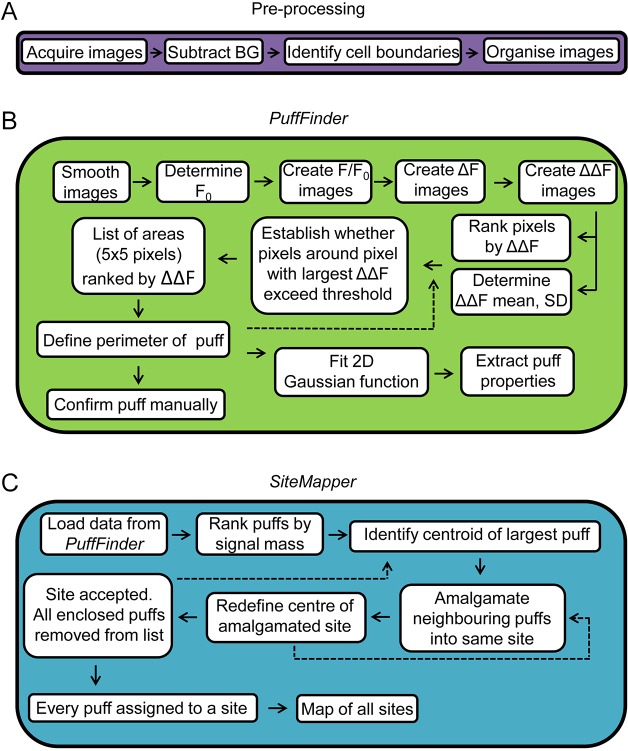
**Automated detection and analysis of Ca^2+^ puffs and sites.** (A) Images are acquired and prepared for analysis by correcting for background (BG) fluorescence, identifying cell boundaries and preparing image stacks for each identified cell. Further details in Materials and Methods. (B) *PuffFinder* is designed to detect Ca^2+^ puffs. Images are smoothed. The pre-stimulus fluorescence values (F_0_) are determined for each pixel. F/F_0_ is then determined for every pixel in every frame. To identify regions where fluorescence changes rapidly, the difference in fluorescence intensity (ΔF) between each image and its immediate successor is determined. *PuffFinder* then corrects these ΔF values for any creeping increase in F/F_0_ by subtracting the average ΔF across every frame from every pixel; this provides the ΔΔF stacks used to identify puffs. The mean and s.d. of the ΔΔF values are determined to provide criteria for identifying puffs. Pixels are ranked by ΔΔF value. The pixel with the largest ΔΔF is placed at the centre of a 5×5 pixel matrix, and selection criteria are applied to decide whether the matrix is an area wherein sufficient pixels have large ΔΔF values. The selection criteria and the rationale for choosing them are elaborated in Materials and Methods. The list of matrices ranked by ΣΔΔF is now interrogated to define the boundaries of each puff. This is achieved by returning to the pixel with the largest ΔΔF value and expanding it outwards until ΔΔF of the enclosed pixels falls below a threshold value. The process is then repeated with the next ranked pixel. The locations of puffs are confirmed by visual inspection and their properties defined after fitting a 2D Gaussian function. (C) *SiteMapper* is used to decide whether Ca^2+^ puffs originate at the same or different sites. It begins with the puffs identified in *PuffMapper* and ranks them according to their signal mass. The centroid of the largest puff is identified and if the centroids of neighbouring puffs fall within 0.96 µm of it, they are amalgamated into the same site. The centre of the new site is defined and the assessment of neighbours is repeated. The site is then accepted and its enclosed puffs are excluded from further analysis. The analysis then moves to the next largest unassigned puff, and the process is repeated until all puffs have been assigned to sites to create a map of all sites in the cell. Further details of *PuffMapper* and *SiteMapper* are provided in the Materials and Methods.

Here, we use TIRFM to image human embryonic kidney 293 (HEK293) cells loaded with a fluorescent Ca^2+^ indicator, and record Ca^2+^ puffs evoked by the photolysis of ci-IP_3_, or by IP_3_ provided by stimulation of endogenous M3 muscarinic acetylcholine receptors with carbachol. By using a custom-written algorithm that allows automated detection of Ca^2+^ puffs and Ca^2+^ release sites (Fig. 1, Figs S1 and S2), we show that both stimuli evoke Ca^2+^ puffs at abundant immobile intracellular sites. We demonstrate that IP_3_ produced in response to stimulation with carbachol, and i-IP_3_ produced by photolysis of ci-IP3 are delivered to the same intracellular Ca^2+^ release sites. Increasing the stimulus intensity causes an increase in the frequency of Ca^2+^ puffs without recruiting additional Ca^2+^ release sites.

## RESULTS

### Extracellular stimuli evoke Ca^2+^ puffs at many intracellular sites

Our aim was to define the spatial distribution of the local Ca^2+^ signals evoked by different stimuli and stimulus intensities. This aim effectively restricts the field of view to ∼82 µm×82 µm, within which there are typically about six HEK293 cells and rarely more than three HeLa cells. It was, therefore, important to establish that the submaximal stimuli that evoke local Ca^2+^ signals stimulate responses in most cells. In populations of HEK293 cells, carbachol evoked Ca^2+^ signals with a half-maximal effective concentration (EC_50_) of ∼40 µM (López-Sanjurjo et al., 2013; Tovey and Taylor, 2013). In single HEK293 cells, maximal (1 mM) and submaximal (10 µM) concentrations of carbachol evoked increases of [Ca^2+^]_c_ in most cells (92±7%, mean±range, *n*=2 fields; and 90±16%, mean±s.e.m., *n*=3 fields, respectively).

**Fig. 2.**
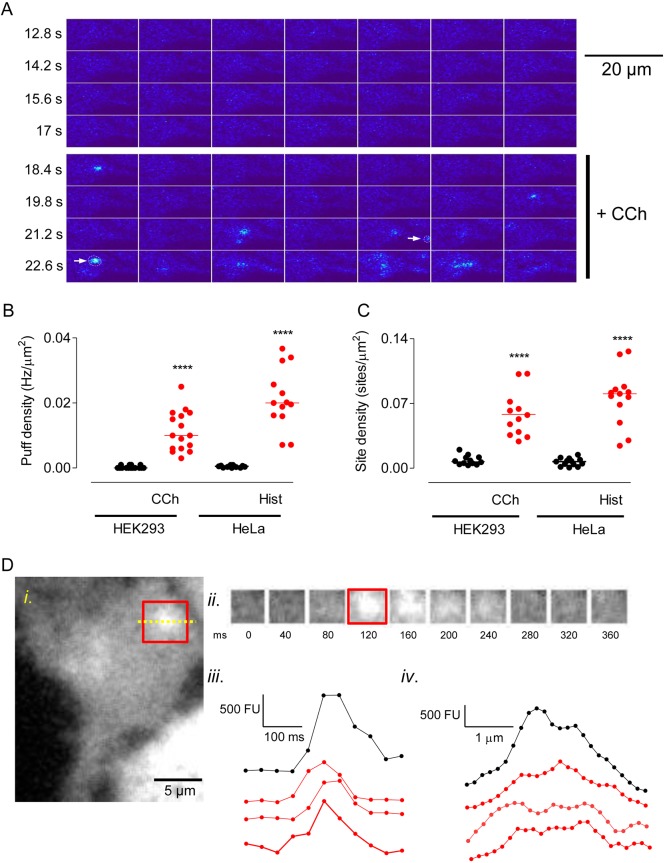
**Extracellular stimuli evoke abundant Ca^2+^ puffs.** (A) Typical TIRFM images from a single Cal520-loaded HEK293 cell, collected at 40-ms intervals, with ΔF shown in pseudocolour at the indicated times before and after addition of carbachol (CCh, 10 µM). Within the montage, images show every 5th frame (i.e. 200 ms between successive frames). Arrows show Ca^2+^ puffs identified by *PuffFinder* (see Fig. S1 for additional examples). (B,C) Summary results show the frequency of Ca^2+^ puffs (Hz/µm^2^) (B) and the density of sites (sites/µm^2^) (C) detected before and after stimulation of HEK293 cells with CCh (10 µM) or HeLa cells with histamine (Hist, 5 µM). Events were recorded for the entire period (up to 10 s) after addition of the stimulus until the Ca^2+^ signal propagated across the cell. Results show the mean and results for individual cells (16 HEK293 cells and 13 HeLa cells). ****P<0.0001, paired Student's *t*-test, relative to unstimulated cells. (D) Example of a Ca^2+^ puff evoked by CCh in a HEK293 cell (*i*). The time course of the fluorescence changes within the boxed area are shown in panels *ii* and *iii*. Images were background-subtracted and smoothed prior to analysis. Panel *iv* shows the fluorescence intensity profile across the line shown within the boxed area. Examples of the time courses (*iii*) and fluorescence intensity profiles (*iv*) of additional Ca^2+^ puffs are shown by red lines. Time courses reflect the average fluorescence intensity of the boxed area (*i*) over time.

Ca^2+^ puffs were very infrequent in unstimulated HEK239 cells, but carbachol (10 µM) evoked a flurry of Ca^2+^ puffs beginning 3.2±0.2 s after carbachol addition (*n*=48, 3 challenges of 16 cells) (Fig. 2A, Fig. S1, Movie 1). Within a few seconds most Ca^2+^ signals then propagated across the cell as a Ca^2+^ wave, as reported for many other cell types (Bootman et al., 1997b; Charles et al., 1991; Marchant et al., 1999; Rooney et al., 1990; Smith et al., 2009a). By using *PuffMapper* to detect Ca^2+^ puffs automatically (see Methods for the criteria used to identify Ca^2+^ puffs), we confirmed the significant increase in the frequency of Ca^2+^ puffs in HEK293 cells stimulated with carbachol and in HeLa cells stimulated with histamine (Fig. 2B). In both cell types, stimulation also significantly increased the number of sites at which Ca^2+^ puffs were observed (Fig. 2C).

Typical examples of individual carbachol-evoked Ca^2+^ puffs in HEK293 are shown in Fig. 2D and Fig. S1B, from which it is clear that the puffs identified by the automated analysis are heterogenous, consistent with results from other cells (Wiltgen et al., 2014). Our methods, designed to optimise spatial resolution (see Materials and Methods), do not provide the high temporal resolution of some studies (e.g. Smith and Parker, 2009). Nevertheless, the temporal and spatial profiles of the local Ca^2+^ signals detected in HeLa and HEK293 cells are consistent with those reported for Ca^2+^ puffs in other cells (Table 1). However, a striking difference is the substantially larger number of puffs and initiation sites detected in our analyses (Table 1). The densities of Ca^2+^ initiation sites (number/µm^2^) detected during a 10-s period of stimulation are similar in HEK293 and HeLa cells (Fig. 2C), but the sites were at least 10-fold more abundant than observed in other studies (Table 1), although none of these studies examined effects of extracellular stimuli on HEK293 cells (Nakamura et al., 2012; Smith and Parker, 2009; Thomas et al., 2000). The unexpectedly large number of Ca^2+^ puffs is unlikely to be due to the automated analysis mistakenly identifying puffs because the temporal and spatial profiles of the Ca^2+^ puffs in HeLa and HEK293 cells are similar to those reported previously (Fig. 2D, Fig. S1B, Table 1). The most likely explanation is that the Ca^2+^ buffering imposed by EGTA in HEK293 and HeLa cells delays the initiation of global Ca^2+^ signals for longer than in other cells, and so extends the opportunity to identify Ca^2+^ puffs and sites. Our automated analysis may also have contributed by detecting events that are missed in manual analyses.

**Table 1.**
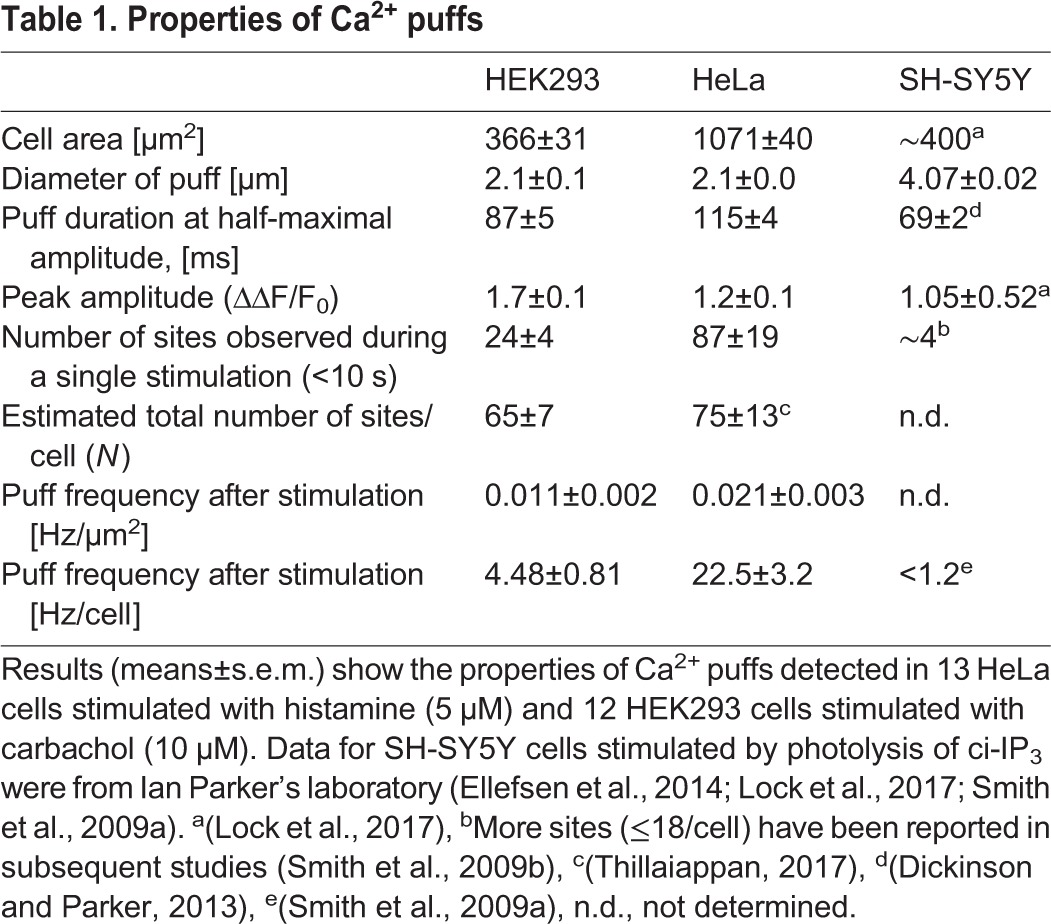
**Properties of Ca^2+^ puffs**

We suggest that Ca^2+^ puffs are more frequent and initiate at many more sites than hitherto observed. That conclusion is consistent with additional analyses of HeLa cells with endogenously tagged IP_3_Rs, where we used a different algorithm (xy-spotter) (Steele and Steele, 2014) to identify Ca^2+^ puffs (Thillaiappan, 2017). Here, too, both histamine and photolysis of ci-IP_3_ evoked frequent Ca^2+^ puffs from relatively abundant sites (Thillaiappan, 2017). Subsequent experiments examine the sites at which Ca^2+^ puffs originate in HEK293 cells.

### Repeated stimulation with carbachol identifies many additional Ca^2+^ release sites

**Fig. 3.**
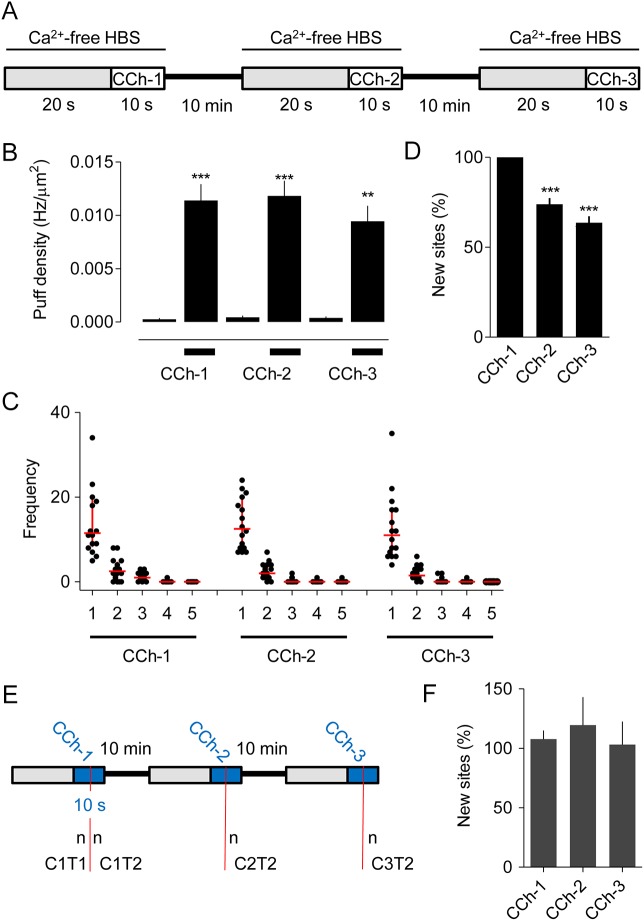
**Carbachol-evoked Ca^2+^ puffs initiate at abundant immobile sites.** (A) The stimulus regime used for repetitive stimulation of Cal520-loaded HEK293 cells with carbachol (CCh, 10 μM). (B) Results show the density of Ca^2+^ puffs (Hz/μm^2^) evoked during each pre-incubation (grey in A) and subsequent stimulation period with CCh. Results (mean± s.e.m.) are from 16 cells. Results for ten individual cells are shown in Fig. S3. ***P<0.001, **P<0.01 relative to pre-incubation values, one-way ANOVA with Bonferroni post hoc test. (C) The number of sites at which 1−5 puffs were detected during the last 2 s after CCh addition for each of the three periods of stimulation. Results show all puffs from 16 cells, with the median and interquartile range shown by horizontal and vertical red lines, respectively. (D) For each cell, the new sites detected during each of three sequential stimulations with CCh are expressed as a percentage of all sites visited during that CCh challenge. Results are means±s.e.m., *n*=16 cells. ***P<0.001 relative to CCh-1, one-way ANOVA with Bonferroni post hoc test. Table S1 shows the data from which this panel was constructed. (E) Analysis used to determine whether Ca^2+^ puffs more effectively discover new release sites when the interval between measurements is extended. See text for details. (F) Summary results show the number of new sites discovered during the T2 phase of the response to each of the three stimulations with CCh (CCh1−3) relative to the sites identified during the T1 phase of the first CCh challenge (C1T1). Hence, a site is reported as new if it was not detected during C1T1. Results (means±s.e.m., *n*=16 cells) are reported as percentages of the number of sites discovered during C1T1.

We investigated whether Ca^2+^ puffs initiate at the same sites during repeated stimulation with carbachol. The optimised protocol, developed to maximise puff frequency and recording intervals while allowing complete recovery between successive stimuli, is shown in Fig. 3A. Basal activity was recorded for 20 s in Ca^2+^-free HBS, followed by addition of carbachol (10 µM) for 10 s; the carbachol was then removed and replaced with normal HBS. After a 10-min recovery period in normal HBS, the medium was replaced with Ca^2+^-free HBS (20 s) and the recording and stimulation sequence was repeated (Fig. 3A, Figs S2 and S3). The results demonstrate that three sequential challenges with the same carbachol concentration evoke the same increase in Ca^2+^ puff activity and with the same frequency distribution of Ca^2+^ puffs across release sites (Fig. 3B,C). Within a few seconds of stimulation with carbachol at concentrations that evoked substantial increases in the frequency of Ca^2+^ puffs, the Ca^2+^ signals usually developed into Ca^2+^ waves that invaded the entire cell. Hence, although the stimulus regime was consistent, the initiation of global Ca^2+^ signals usually and unpredictably terminated our ability to resolve Ca^2+^ puffs before the end of the 10-s incubation period with carbachol. Others have also reported difficulties in recording Ca^2+^ puffs evoked by extracellular stimuli without rapidly triggering Ca^2+^ waves (Smith et al., 2009a). In all our analyses of responses to multiple challenges (carbachol or ci-IP_3_), the shortest interval between detection of the first Ca^2+^ puff and initiation of a global Ca^2+^ signal was 2 s. In subsequent quantitative analyses we, therefore, matched the analysis interval for all recordings by analysing puff activity during only the last 2 s before either initiation of the global Ca^2+^ signal or termination of the carbachol exposure.

The results so far suggest that Ca^2+^ puffs initiate at many sites but, even this, may underestimate their number because brief periods of recording may detect too few puffs to reveal the locations of all sites. We would then expect each carbachol challenge to discover new sites, but fewer with each successive stimulus. Fig. 3D confirms this prediction. During the second stimulation with carbachol, for example, only 26±3% (*n*=16) of the active Ca^2+^ release sites had also responded during the first challenge (Fig. 3D, Table S1). These results suggest that the second challenge, during which 17±1.8 sites/cell were identified (Table S1), reveal only 26% of the sites capable of initiating Ca^2+^ puffs. From this, we estimate the total number of Ca^2+^ release sites in a cell (*N*) to be 65±7. An alternative approach uses the Poisson distribution to estimate the mean number of puffs/site (µ) and then estimates *N* from µ (Table S2). This Poisson analysis applied to the first carbachol stimulation suggests that there are 70±13 sites/cell. The two analyses are consistent in suggesting that there are ∼70 carbachol-sensitive Ca^2+^ release sites/cell. Henceforth, we estimate *N* from comparisons of responses to the first and second stimuli because it is less prone than the Poisson analysis to errors arising from the small numbers of sites that respond twice during our short analysis intervals (Table S2).

**Fig. 4.**
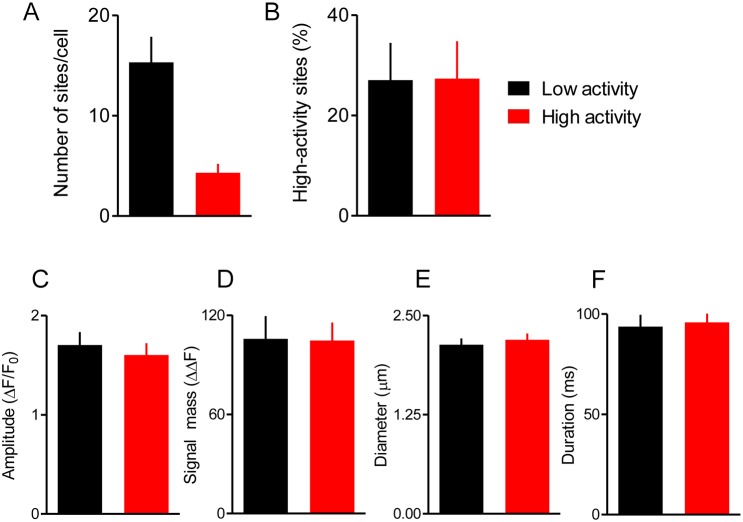
**Most Ca^2+^ release sites have similar properties.** (A,B) Sites at which Ca^2+^ puffs occurred during the first challenge with carbachol (Fig. 3A) were categorised as ‘low-activity’ if they responded with a single Ca^2+^ puff and ‘high-activity’ if they responded with several puffs. Summaries show the numbers of each category of site (A) and the percentage of sites evoking more than one Ca^2+^ puff during subsequent challenges with carbachol according to whether the sites were categorised as high- or low-activity during the first challenge (B). Results are means±s.e.m. from 16 cells. (C-F) Summary data shows puff amplitudes (C), signal mass (D), puff diameters (E) and durations at half-maximal amplitude (F) for puffs evoked at each category of site. There were no significant differences (paired Student's *t*-test) in the properties of Ca^2+^ puffs between low- and high-activity sites. Puff amplitude is defined as the maximum ΔΔF value within the puff region. Signal mass is defined as ΣΔΔF for pixels within the puff region that are ≥T2 (see Materials and Methods). Puff diameters are the average of the major and minor elliptical diameters from the 2D Gaussian fit to the puff region. Results are means±s.e.m. from 16 cells responding to carbachol (10 μM).

In other cells, it has been concluded that there may be eager and reluctant Ca^2+^ release sites (Dickinson and Parker, 2013; Rooney et al., 1990; Smith et al., 2009a; Thomas et al., 2000), but our estimates of *N* assume that each site has a similar probability of initiating a Ca^2+^ puff. To test this assumption, we first categorised sites as low- or high-activity according to whether they responded once or more than once during the first exposure to carbachol; 78% of sites were categorised as low-activity (Fig. 4A). We then asked how Ca^2+^ puffs are distributed across the two categories of sites during subsequent responses to carbachol. The results show that the fraction of sites responding with more than one Ca^2+^ puff during the second and third carbachol challenges was the same for low- and high-activity sites (Fig. 4B). Furthermore, there were no significant differences in the properties of Ca^2+^ puffs at sites that responded once or several times during each carbachol challenge (Fig. 4C-F). We conclude that, in HEK293 cells, there is no evidence for any systematic difference between sites that respond with multiple Ca^2+^ puffs and those that respond only once.

We obtained matched records from all cells for quantitative analysis by selecting only 2 s from each 10-s recording for analysis, but at the expense of having to extrapolate data to estimate the total number of Ca^2+^ release sites (*N*). During the 6 s of stimulation used for analysis of the three sequential carbachol challenges, 41±5 distinct sites/cell (*n*=16 cells) were directly identified, whereas our extrapolated analyses suggest 65±7 sites/cell. Hence, both the number of directly identified sites and the extrapolated estimate of *N* suggest that Ca^2+^ release sites are more abundant than hitherto suggested. We conclude that carbachol evokes Ca^2+^ release from a large number of distinct sites in HEK293 cells.

### Ca^2+^ release sites are immobile

The large number of sites at which Ca^2+^ puffs initiate and the frequency with which new sites are discovered during successive stimulations with carbachol (Fig. 3A-D) suggest that there are either many more sites than can be discovered during brief periods of carbachol stimulation or that sites move during the 10-min intervals between stimulations (Fig. 3A). The consistent estimates of *N* from analyses that compare Ca^2+^ puffs within (70±13 sites/cell, Table S2) and between (65±7, Table S1) carbachol challenges suggest that movement of sites is probably not a major factor. Nevertheless, to distinguish between the two possibilities we used the method shown in Fig. 3E to compare the rate of discovering new Ca^2+^ release sites within a short (<10 s) stimulation with carbachol to the rate of discovery after a 10-min or 20-min recovery period. For each cell, all Ca^2+^ puffs during the first carbachol stimulation were counted before the recording was terminated at either 10 s or by initiation of a Ca^2+^ wave. This first period of stimulation (C1) was then split to provide two intervals (T1 and T2), each with an equal number of Ca^2+^ puffs (*n*). For the same cell, we then examined the second (C2) and third (C3) response to carbachol and identified the interval (T2) during which *n* Ca^2+^ puffs occurred (Fig. 3E). This allowed comparison, in each cell and for matched numbers of Ca^2+^ puffs, of the rates of discovery of new Ca^2+^ release sites under conditions where the comparison spanned <10 s (C1T1 versus C1T2), 10 min (C1T1 versus C2T2) or 20 min (C1T1 versus C3T2). For the latter, sites have more than 120-times longer to move than during the first comparison (CIT1 versus C1T2). The results demonstrate that the Ca^2+^ puffs discover new sites at similar rates whether the comparisons span a few seconds or more than 20 min (Fig. 3F).

These results suggest that the sites that respond to carbachol are immobile for many minutes, consistent with previous analyses of SH-SY5Y cells (Smith et al., 2009b) and with recent observations showing that only immobile IP_3_Rs respond to IP_3_ (Thillaiappan, 2017). However, we suggest that there are many more of these Ca^2+^ release sites (65±7 sites/cell) than hitherto supposed (Table 1). Since we record Ca^2+^ puffs only within the TIRF field (i.e. within ∼62 nm of the basal plasma membrane), we may underestimate the total number of sites in a cell. However, both we for HeLa cells (see Thillaiappan, 2017) and others for SH-SY5Y cells (Smith et al., 2009a) have shown that most IP_3_-evoked Ca^2+^ puffs occur within the TIRF field, suggesting that our recordings probably capture most Ca^2+^ release sites.

### Photolysis of ci-IP_3_ also reveals many Ca^2+^ release sites

**Fig. 5.**
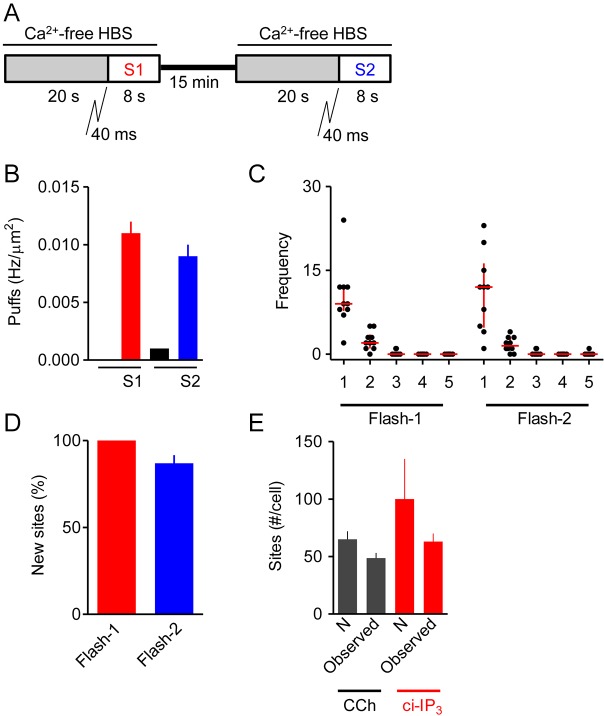
**Responses to repeated photolysis of ci-IP_3_.** (A) HEK293 cells loaded with ci-IP_3_ were exposed to a 40-ms UV flash at the beginning of each of the two stimulation intervals (S1 and S2). (B) Results show the density of Ca^2+^ puffs (Hz/μm^2^) evoked during each pre-incubation (grey in A) and after each photolysis of ci-IP_3_. There was no significant difference between the responses evoked by the first and second flashes. (C) The number of sites at which 1−5 puffs were detected during the last 2 s of the 8-s recording after photolysis of ci-IP_3_ for each of the two periods of stimulation. Results show all puffs from 10 cells, with the median and interquartile range shown by red lines. (D) For each cell, the new sites detected during each of two sequential UV flashes to photolyse ci-IP_3_ are expressed as a percentage of all sites visited during that stimulation. Results are means±s.e.m., *n*=10 cells. Table S3 shows the data from which this panel was constructed. (E) Summary results (mean±s.e.m.) show numbers of Ca^2+^- release sites identified directly from counting all Ca^2+^ puffs during all stimulations with carbachol (3×10 s, 16 cells) or photolysis of ci-IP_3_ (3×8 s, 10 cells) (Observed), or by estimating *N* as described in Tables S1 and S3.

We next examined the distribution of Ca^2+^ puffs evoked by uniformly delivering the active IP_3_ analog d-2,3-*O*-isopropylidene-IP_3_ (i-IP_3_) to the cytosol by photolysis of ci-IP_3_. Photolysis of ci-IP_3_ evoked a flurry of Ca^2+^ puffs after a latency of 0.42±0.09 s (Movie 2). We used a stimulus regime similar to that used for sequential stimulation with carbachol, but with the recording period reduced to 8 s (to match the 8 s available for recording after the ∼2 s taken to add carbachol), and with the recovery period extended to 15 min (since i-IP_3_ is less rapidly metabolised than IP_3_) (Fig. 5A). It proved impossible to get matched responses to all three rounds of photolysis of ci-IP_3_ with complete recovery between them (Fig. S4). The incomplete recovery is probably due to i-IP_3_ being less rapidly degraded than IP_3_ (Dakin and Li, 2007), and the diminished responses to successive flashes are likely due to depletion of ci-IP_3_ with each flash. Despite the limitations, the optimised photolysis protocol achieved matched Ca^2+^ puff activity to two successive UV flashes (Fig. 5B,C, Table S3). We again analysed only the 2 s immediately preceding either initiation of a global Ca^2+^ signal or termination of the 8-s recording interval and used the rate of discovery of new sites between successive challenges to estimate the total number of Ca^2+^ release sites/cell (*N*=100±35, *n*=10 cells) (Fig. 5D, Table S3). Counting all distinct Ca^2+^ release sites detected throughout the three 8-s recordings after photolysis of ci-IP_3_ (mean recording interval 23±0.2 s) identified 63±7 sites/cell (Fig. 5E). There is no significant difference in the estimated numbers of Ca^2+^ release sites accessible to carbachol (65±7 sites/cell) and to i-IP_3_ delivered uniformly to the cytosol (100±35). We conclude that both carbachol and photolysis of ci-IP_3_ evoke Ca^2+^ release from a large number of immobile sites in HEK293 cells (Fig. 5E).

### Increasing the carbachol concentration does not reveal new Ca^2+^ release sites

**Fig. 6.**
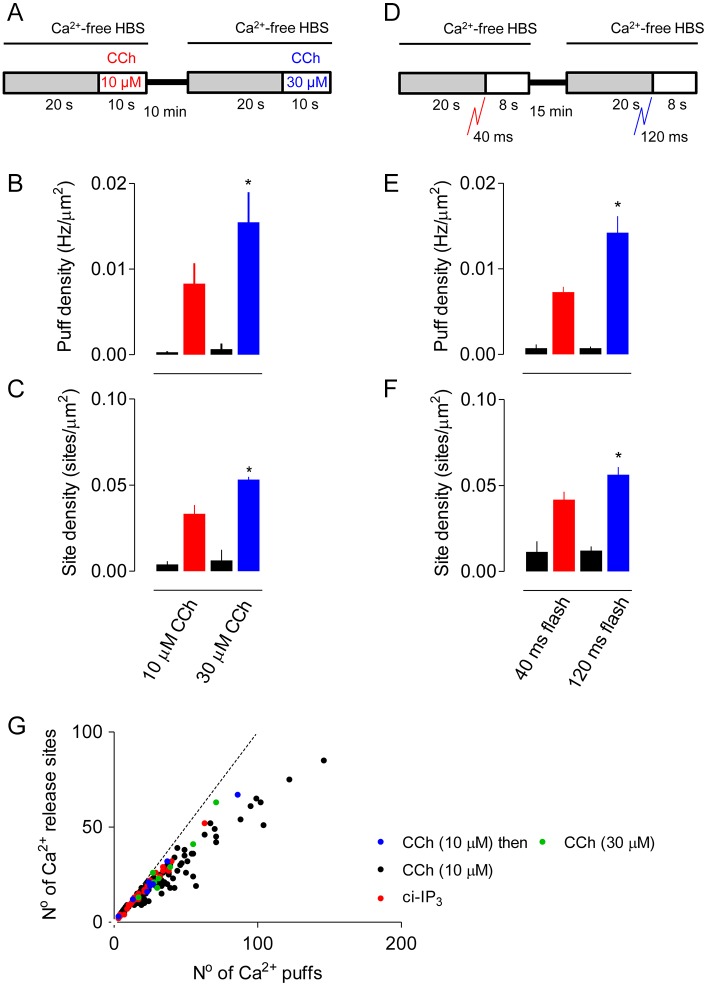
**Increasing carbachol concentration increases the frequency of Ca^2+^ puffs, but not the number of sites.** (A) Cells were sequentially stimulated with two concentrations of carbachol (CCh). (B,C) Summary results show the frequency of Ca^2+^ puffs (B) and the number of sites detected (C) before and after stimulation with the indicated CCh concentrations. Events were recorded for the entire period (up to 10 s) after addition of CCh until the Ca^2+^ signal propagated across the cell. Results (mean±s.e.m.) are from 8 cells. *P<0.05, paired Student's *t*-test, relative to first stimulus. (D) Cells were stimulated with two sequential UV flashes to photolyse ci-IP_3_. (E,F) Summary results show the frequency of Ca^2+^ puffs (E) and the number of sites detected (F) before and after each stimulation. Results (mean±s.e.m.) are from 10 cells. *P<0.05, paired Student's *t*-test, relative to first stimulus. (G) Relationship between number of Ca^2+^ puffs/cell and the unique Ca^2+^ release sites they identify for cells stimulated once or repetitively with CCh (10 µM, black) or by photolysis of ci-IP_3_ (40 ms, red). The responses to sequential stimulation with 10 µM (blue) and then 30 µM CCh (green) are shown for 8 cells. The dashed line shows the relationship if every Ca^2+^ puff identified a new Ca^2+^ release site.

To examine the effects of varying stimulus intensity, HEK293 cells were first stimulated for ∼10 s with 10 µM carbachol and then with 30 µM carbachol according to the protocol shown in Fig. 6A. The results demonstrate that the cells fully recovered between the two periods of stimulation, and that increasing the carbachol concentration increased the frequency of Ca^2+^ puffs and the number of sites detected (Fig. 6B,C). Similar results were obtained with ci-IP_3_ when the duration of the flash used for photolysis was increased (Fig. 6D-F).

The increase in frequency of Ca^2+^ puffs with carbachol concentration might be due to an increased probability of every Ca^2+^ release site responding or, more-intense stimulation might increase the number of sites competent to respond. The first explanation predicts that a fixed number of Ca^2+^ puffs should discover the same number of Ca^2+^ release sites whatever the stimulus intensity, whereas the second explanation predicts that Ca^2+^ puffs evoked by the high carbachol concentration should more effectively discover new Ca^2+^ release sites. We, therefore, compared the relationship between the number of Ca^2+^ puffs and the number of unique sites they reveal for cells stimulated with low and high carbachol concentrations. The results demonstrate that the relationship between Ca^2+^ puffs and the number of Ca^2+^ release sites they identify is the same for carbachol and photolysis of ci-IP_3_ and, furthermore, it is the same for low and high concentrations of carbachol (Fig. 6G).

These results demonstrate that a Ca^2+^ puff has the same probability of discovering a Ca^2+^ release site, regardless of whether the Ca^2+^ signals are evoked by carbachol or photolysis of ci-IP_3_, or are evoked by low or high stimulus intensities. We conclude that the increased frequency of Ca^2+^ puffs as stimulus intensity is increased results from most sites becoming more likely to respond rather than from recruitment of new Ca^2+^ release sites.

### Carbachol and photolysis of ci-IP_3_ activate the same Ca^2+^ release sites

**Fig. 7.**
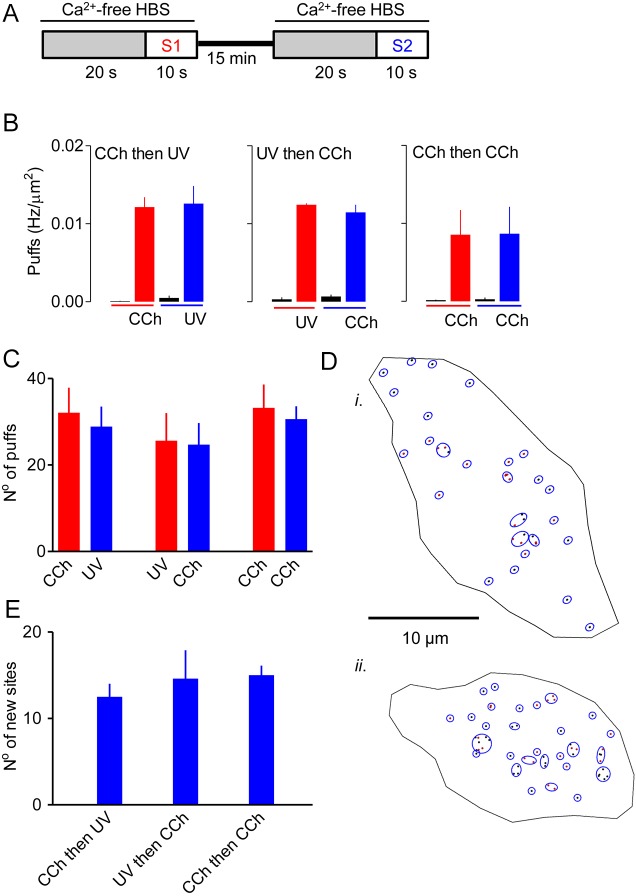
**Carbachol and photolysis of ci-IP_3_ stimulate the same Ca^2+^ release sites.** (A) Cells loaded with ci-IP_3_ were sequentially stimulated with carbachol (10 μM) and a UV-flash (120 ms), with either stimulus presented first. (B) Density of Ca^2+^ puffs evoked by the two stimuli presented in either order during the entire 10-s recording interval for each stimulus. The activity recorded before each stimulus is also shown. Results show no significant differences between any of the stimuli. (C) Summary results, from analyses of the entire period of stimulation preceding initiation of a Ca^2+^ wave show the number of Ca^2+^ puffs detected during each of the indicated pairs of sequential stimuli. (D) Typical results show centroids of the Ca^2+^ puffs detected by *PuffFinder* during stimulation first with carbachol (10 µM, black) and then with ci-IP_3_ (120 ms, red) (*i*) or with the same stimuli in reverse order (*ii*). Sites detected by *SiteMapper* are shown by blue circles. (E) Summary results show the number of new Ca^2+^ release sites identified during the second stimulus (S2) that were not detected during the first period of stimulation (S1) for each of the indicated pairs of stimuli. Results (C and E) show means±s.e.m. from 10−18 cells.

We next considered whether i-IP_3_ delivered uniformly to the cytosol by photolysis of ci-IP_3_ or IP_3_ delivered from endogenous signalling pathways evokes Ca^2+^ puffs at the same sites. Cells loaded with ci-IP_3_ were sequentially stimulated with carbachol (10 μM) and a photolysis flash (120 ms) (Fig. 7A). The two stimuli evoked indistinguishable Ca^2+^ puff activity irrespective of the order in which they were presented (Fig. 7B,C). We then asked whether, with these matched stimulus intensities, the second stimulus from a heterologous sequence of stimuli identified more new Ca^2+^ release sites than the second stimulus from two sequential carbachol challenges. The results show that the number of new sites identified by the second stimulus was unaffected by the nature of the first stimulus (Fig. 7D,E). The results also suggest that the same Ca^2+^ release sites are activated by endogenous signalling pathways and by photolysis of ci-IP_3_.

## DISCUSSION

We used a custom-written algorithm that automatically detects Ca^2+^ puffs and assigns them to Ca^2+^ release sites in order to examine the spatial organisation of IP_3_-evoked Ca^2+^ signals in human cell lines (Figs 1 and 2, Figs S1 and S2). In both HEK293 and HeLa cells, stimuli that evoke formation of IP_3_ through endogenous G-protein-coupled receptors (GPCRs) triggered a flurry of Ca^2+^ puffs whose properties were similar to those of Ca^2+^ puffs observed in other cells (Table 1, Fig. 2D, Fig. S1B). However, the sites at which these Ca^2+^ puffs occurred were about 10-fold more abundant than reported previously for other cells (Nakamura et al., 2012; Smith and Parker, 2009; Thomas et al., 2000). In HEK293 cells, for example, we estimate the number of sites to be 65±7 sites/cell (Fig. 5E; Table S1), and our less-comprehensive analysis of HeLa cells suggests at least 87 sites/cell (Table 1). The latter is consistent with our recent, independent analysis of Ca^2+^ puffs in HeLa cells that express endogenous IP_3_Rs tagged with EGFP, wherein photolysis of ci-IP_3_ evoked many Ca^2+^ puffs at numerous sites (see Thillaiappan, 2017). We conclude that Ca^2+^ release sites are more abundant than hitherto suggested but, even so, with about eight IP_3_Rs per Ca^2+^ release site (see Thillaiappan, 2017) and about 7200 IP_3_Rs per HEK293 cell (Tovey et al., 2008), it is clear that only a small fraction of IP_3_Rs (<8%) contribute to the Ca^2+^ release sites.

Many studies have shown that IP_3_Rs can move within ER membranes (see references in Geyer et al., 2015); yet the Ca^2+^ puffs evoked by stimulation of cells with low stimulus intensities are often observed to initiate at immobile sites (Bootman et al., 1997a; Kasai et al., 1993; Marchant and Parker, 2001; Nakamura et al., 2012; Olson et al., 2012; Rooney et al., 1990; Simpson et al., 1997). We confirmed those observations by demonstrating that, in HEK293 cells, Ca^2+^ puffs initiate at sites that remain immobile for at least 20 min (Fig. 3E,F). Among these immobile Ca^2+^ release sites, we found no evidence to suggest any systematic difference in their propensity to initiate Ca^2+^ signals (Fig. 4). We suggest, in keeping with the conclusions of our recent analyses of HeLa cells (Thillaiappan, 2017), that Ca^2+^ puffs initiate at immobilised IP_3_R clusters that comprise only a small fraction of the IP_3_Rs expressed in each cell.

Comparison of the effects of sequential challenges with different carbachol concentrations confirmed that Ca^2+^ puffs become more frequent as the stimulus intensity increases, with no apparent increase in the number of Ca^2+^ release sites (Fig. 6). From this, we conclude that more-intense stimulation increases the probability of a site initiating a puff without increasing the underlying number of competent Ca^2+^ release sites.

By comparing sequential responses of cells to photolysis of ci-IP_3_ and IP_3_ delivered to the cell through an endogenous GPCR signalling pathway, we established that each stimulus evoked Ca^2+^ puffs from the same immobile Ca^2+^ release sites (Fig. 7). This result suggests that global delivery of i-IP_3_ and IP_3_ provided at the plasma membrane by hydrolysis of phosphatidylinositol 4,5-bisphosphate by GPCR-activated PLC reach the same IP_3_Rs. While our work was in progress, a study of SH-SY5Y cells reached a similar conclusion, namely that the sites where Ca^2+^ puffs initiate are probably the same, regardless of whether the signals are evoked by photolysis of ci-IP_3_, or by carbachol or bradykinin activating their respective GPCRs (Lock et al., 2017). These results are unexpected because there is evidence that some GPCRs may selectively deliver IP_3_ to IP_3_Rs (Delmas et al., 2002; Olson et al., 2012; Short et al., 2000; Tovey and Taylor, 2013; Tu et al., 1998; Zhao et al., 1990), and both we (Taylor and Konieczny, 2016) and others (Dickinson et al., 2016) have suggested that IP_3_Rs substantially slow diffusion of IP_3_ within cells. A possible explanation is that the only IP_3_Rs that contribute to initiation of Ca^2+^ puffs are those that lie very close to the plasma membrane (Smith et al., 2009a; Thillaiappan, 2017). In this situation, even if IP_3_ were locally delivered from GPCR signalling pathways we might fail to distinguish responses evoked by GPCRs from those evoked by uniform photolysis of caged IP_3_ since the only responsive IP_3_Rs would be those located close to the plasma membrane.

Our quantitative analyses of the spatial distribution of Ca^2+^ puffs, the building blocks of all IP_3_-evoked Ca^2+^ signals, has established that Ca^2+^ puffs initiate at relatively abundant immobile sites (∼65 sites/cell) that include only a small fraction of the IP_3_Rs expressed in a cell. The number of sites appears not to be affected by the intensity of the stimulation, and there is no evidence to suggest that IP_3_ is selectively delivered to them from endogenous signalling pathways.

## MATERIALS AND METHODS

### Materials

Cal520-AM was from AAT Bioquest (Sunnyvale, CA). Fluo-4-AM, Dulbecco's Modified Eagle's Medium (DMEM)/Ham's F-12 (50:50) supplemented with GlutaMAX, and TrypLE were from Invitrogen (Paisley, UK). Pluronic acid F-127, dimethyl sulfoxide (DMSO), poly-L-lysine, foetal bovine serum (FBS), histamine and carbamylcholine chloride (carbachol) were from Sigma-Aldrich (Poole, Dorset, UK). Fibronectin and EGTA-AM were from Merck Millipore (Darmstadt, Germany). Caged IP_3_-PM [ci-IP_3_-PM, d-2,3-*O*-isopropylidine-6-*O*-(2-nitro-4,5-dimethoxy)benzyl-*myo*-inositol 1,4,5-trisphosphate-hexakis(proprionoxymethyl)ester] was from SiChem GmbH (Bremen, Germany).

### Cell culture

We used two cell lines, HEK293 and HeLa cells (ATCC), neither of which express functional ryanodine receptors (RyRs). There is detectable mRNA for RyRs in HeLa cells, but caffeine does not evoke Ca^2+^ signals (Tovey et al., 2001). HEK293 cells do not express RyRs, and are insensitive to caffeine and ryanodine (López-Sanjurjo et al., 2013; Tong et al., 1999). HEK293 and HeLa cells were maintained in DMEM/Ham's F-12 supplemented with GlutaMAX and 10% FBS in humidified air at 37°C with 5% CO_2_. The cells were passaged at ∼80% confluence. Short tandem-repeat profiling was used to authenticate HeLa cells (Eurofins, Germany) and HEK293 cells (Public Health England). Regular screening confirmed that all cells were free of mycoplasma infection. Cells were grown on either round glass coverslips for low-resolution measurements (No. 1, 22-mm diameter, Scientific Laboratory Supplies, East Yorkshire, UK) or glass-bottomed dishes for high-resolution measurements (No. 1, 35-mm diameter, MaTek, Ashland, MA). The glass was coated with fibronectin (10 µg/ml) for HeLa cells or poly-l-lysine (10 µg/ml) for HEK293 cells. Coated coverslips in 6-well culture plates were seeded with HEK293 cells (8×10^5^ cells/well) or HeLa cells (9×10^4^ cells/well) and used for imaging after 48 h, when they were still subconfluent.

### Imaging of cytosolic Ca^2+^ signals

To allow recording of local Ca^2+^ signals, cells were loaded with EGTA to restrict Ca^2+^ diffusion (Parker and Smith, 2010), a Ca^2+^ indicator (Cal520) and, in some cases, with ci-IP_3_. Cells were washed with HEPES-buffered saline (HBS: 135 mM NaCl, 5.9 mM KCl, 1.2 mM MgCl_2_, 1.5 mM CaCl_2_, 11.5 mM glucose, 11.6 mM HEPES pH 7.3) and then incubated in darkness with pluronic acid F-127 (0.02% w/v) and either ci-IP_3_-PM (1 µM) or DMSO (0.02% v/v) at 20°C. After 45 min, Cal520-AM (2 µM) was added and after a further 45 min the medium was replaced with fresh HBS supplemented with EGTA-AM (10 µM). Cells were used for imaging after a further 45 min. Similar loading methods, but without EGTA-AM or ci-IP_3_-PM, and with Fluo-4 as the Ca^2+^ indicator, were used for low-resolution measurements of [Ca^2+^]_c_.

Local Ca^2+^ signals were measured using total internal reflection fluorescence microscopy (TIRFM). A glass-bottomed dish containing loaded cells was immobilised on the motorised stage of an Olympus IX83 inverted microscope. Imaging used a 100× TIRF objective [Olympus UApo N; numerical aperture (NA)=1.49] with excitation (488 nm) provided by a diode-pumped solid-state laser (150 mW, iLas Laser System, Cairn) and a band-pass filter (ET-405/488/561/643 quad band filter set, Chroma). The angle of the excitation beam was adjusted to achieve TIRF with a penetration depth of ∼62 nm. Emitted light was captured through a band-pass filter (510−550 nm, Olympus U-MNIBA3 cube) with an Andor iXon 897 EMCCD camera (512×512 pixels, 16 µm×16 µm per pixel). With the 100× objective, each image pixel had dimensions of 160 nm×160 nm. Images were acquired at 25 Hz.

Cells were kept in HBS (2 ml, 20°C) in the intervals between stimulation. The HBS was replaced by Ca^2+^-free HBS immediately before recordings. Extracellular stimuli were applied in Ca^2+^-free HBS after careful aspiration of half the medium and its replacement with histamine or carbachol at twice its final concentration in Ca^2+^-free HBS (1 ml). Medium exchanges took ∼1−2 s. Photolysis of ci-IP_3_ was achieved using a light-emitting diode (LED, 395 nm, Spectra Lumincor, Beaverton, OR), with the duration of exposure determined by a shutter controlled from within MetaMorph (Molecular Devices, Sunnyvale, CA). A 50:50 mirror in the excitation light path allowed simultaneous excitation with the 488-nm laser (Cal-520) and the 395-nm LED (for photolysis of ci-IP_3_). Images were captured in Metamorph and saved as tif files for analysis.

Similar methods were used for low-resolution measurements of [Ca^2+^]_c_ in cells grown on glass coverslips, loaded with Fluo-4, and mounted in a stainless steel chamber (∼1 ml) on the stage of an Olympus IX81 inverted microscope with a 40× objective (Olympus UApo N340, NA=1.35). Fluo-4 was excited (488 nm) by light from a diode-pumped solid-state laser (Olympus, Las/405/50) and a band-pass filter (470−490 nm, Semrock, BrightLine Triple Cube). Emitted light was captured with an Andor iXon 897 EMCCD camera (each image pixel had dimensions of 400 nm×400 nm) after passing through a band-pass filter (510−550 nm, Olympus U-MNIBA3 cube). Images (45-ms capture time) were acquired at 2 Hz.

### Automated detection of Ca^2+^ puffs by using *PuffFinder*

The need for automated detection of Ca^2+^ puffs and the unsuitability of the algorithms developed for detection of local Ca^2+^ signals evoked by RyRs have been discussed by Parker and colleagues (Ellefsen et al., 2014). For our automated analyses, images (in tif, tagged image format) from a complete experiment were compiled into a single stack. The images were corrected for background fluorescence by subtracting from every pixel the average fluorescence intensity from a square (∼40 µm^2^) region of interest (ROI) outside the cell, determined uniquely for every frame. Cell boundaries were identified by comparison with differential interference contrast images, and fluorescence values within the extracellular regions were set to zero. ROIs corresponding to each cell were then distributed to separate image stacks: one image stack for each cell. A custom-written suite of algorithms (*PuffMapper*) written in IDL code was used to automatically identify Ca^2+^ puffs (*PuffFinder*) from the background-corrected image stacks for each cell and to then allocate Ca^2+^ puffs to puff sites (*SiteMapper*) (Fig. 1, Figs S1 and S2).

Fig. 1 summarises the steps used by *PuffFinder* to identify Ca^2+^ puffs. The IDL code is available from M.V.K. *PuffFinder* begins by smoothing the background-corrected images across areas corresponding to 2×2 pixels using the ‘Smooth’ function in IDL. This step aids the initial identification of puffs. The unsmoothed images are retained for subsequent analysis of the Ca^2+^ puffs once their locations have been identified. The average fluorescence intensity of each pixel (F_0_) is calculated from 50 pre-stimulation frames. For every subsequent frame, F/F_0_ is calculated for every pixel (F is the fluorescence intensity at each time). The fluorescence change associated with a Ca^2+^ puff typically rises very quickly (within ∼40−60 ms) to its peak value (Nakamura et al., 2012; Smith and Parker, 2009). Our recording interval (40 ms) was chosen to maximise the possibility that this rising phase would occur within a single frame and so allow puffs to be detected by searching for rapid frame-to-frame increases in fluorescence intensity. *PuffMapper* searches for these changes by creating an image stack in which each frame in the F/F_0_ stack is subtracted from the next frame in the temporal series to create a ΔF stack [ΔF=(F_*n*+1_/F_0_)−(F_*n*_/F_0_)]. As stimulation proceeds, Ca^2+^ puffs become more frequent, the background [Ca^2+^]_c_ increases and, eventually, a global Ca^2+^ signal initiates (Movies 1 and 2). To allow detection of Ca^2+^ puffs against this creeping (and then explosive) increase in F/F_0_, *PuffFinder* calculates the average ΔF value across the entire cell for each frame (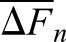). This value is negligibly influenced by the appearance of a Ca^2+^ puff within the frame because Ca^2+^ puffs are relatively rare and each occupies only a small fraction of the cell area (typically ∼0.2%). The 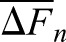 value is subtracted from the ΔF value in each pixel of the same frame (Δ*F*_*n*_) to create a new image stack (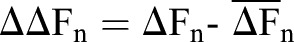). In this ΔΔF stack, the average value of all pixels is zero, but those in which the fluorescence increases rapidly above the prevailing global fluorescence intensity have positive values. *PuffFinder* now scrutinises these ΔΔF values to determine which pixels are likely to be at the centre of a rapid fluorescence increase that extends radially to neighbouring pixels. Since missed events are likely to cause fewer problems in subsequent analyses than mistakenly identified puffs, the algorithm was developed to minimise inclusion of such ‘false-positives’.

Pixels at the centre of a puff will have the largest ΔΔF values, while surrounding pixels will have lower values. *PuffFinder* identifies these pixels using two threshold values (T1 and T2). For each pixel, the mean ΔΔF value (µ, invariably close to 0) and the standard deviation (s.d.) are determined over time. The thresholds (determined empirically) are set at T1=µ+(3.4×s.d.), and T2=µ+(2.45×s.d.), to provide two additional frames (T1 and T2). Every frame in an image stack is now compared with the T1 frame to identify all pixels that exceed T1. Since many of these pixels will be part of the same Ca^2+^ puff, the next step is to identify the pixel that is likely to be at the centre of a puff by finding the pixel with the largest ΔΔF and surrounded by pixels with the largest ΣΔΔF. This is achieved by ranking pixels in each frame in descending order of ΔΔF and by considering the largest first before progressing to the next ranked pixel (excluding those that have already been assigned to a puff). The first pixel is placed at the centre of a 5×5 binary matrix, and each of the 25 pixels is then assigned a binary value (V): V=1 if ΔΔF>T2, and V=0 if ΔΔF≤T2. We chose a 25-pixel area for this analysis because its width (800 nm) would be expected to capture the core of most Ca^2+^ puffs (diameter 2.1±0.1 μm, Table 1). Matrices are then ranked by their ΣV values (ΣΔΔF for all pixels with ΔΔF>T2, the ‘signal mass’ is used as a tie-breaker for matrices with the same ΣV). The final criterion applied to decide whether a pixel forms part of a puff is to eliminate matrices in which <18 pixels exceed T2 (this was again determined empirically). The output from this analysis is a ranked list for each frame of regions (5×5 pixels) with the largest ΔΔF values.

The next step defines the boundary of each puff, beginning with a 3×3 matrix centred on the initial pixel with the largest ΔΔF value. This is achieved by first expanding each matrix to the left and to the right, incrementally one column at a time, until the number of pixels in the column where ΔΔF>T2 falls to zero. The process is now repeated on this wider matrix by expanding rows incrementally up and then down, until the number of pixels within a row in which ΔΔF>T2 falls below 10%. The matrix delineated by this first round of expansion in 2 dimensions is now re-examined, and expanded outwards (columns then rows) to include only columns or rows in which more than 10% of pixels have ΔΔF>T2. The process is then repeated on the next ranked matrix for which the central pixel has not already been placed within an expanded matrix. The output from this processing is a rectangular map enclosing each puff event. The reliability of *PuffFinder* was validated by confirming that all automatically identified puffs could be resolved by manual inspection of fluorescence time series.

The ΔΔF values within each rectangular map of a puff are now fitted to a 2-dimensional Gaussian fit using the GAUSS2FIT function in IDL. This provides the radii of the elliptical fit, its rotation angle (from which the area of the puff is calculated), and the coordinates of the centre of the puff with sub-pixel resolution. Comparison of these features across sequential ΔΔF frames provides temporal properties and the maximal amplitude of the puff.

### Automated identification of Ca^2+^ puff sites by using *SiteMapper*

The second major component of the automated analysis uses *SiteMapper* to identify the different sites at which Ca^2+^ puffs occur (Fig. 1C). The algorithm defines a minimal separation between the centroids of all Ca^2+^ puffs (radius=0.96 µm, i.e. 6 pixels) within a time series to decide whether two puffs occurred at different sites. A similar proximity-based option is provided in the automated analyses described by Parker's group (Ellefsen et al., 2014). The choice of minimal radius is critical for defining the number of sites identified. It was chosen because the average elliptical radii from the 2D Gaussian fits to Ca^2+^ puffs in HEK293 cells was 1.1±0.1 µm (Table 1) and by empirically comparing the relationship between Ca^2+^ puffs and sites after selecting different minimal radii.

*SiteMapper* begins by ranking each Ca^2+^ puff identified by *PuffFinder* throughout a time series according to its signal mass. The first site is initially defined as the centroid of the puff with the greatest signal mass. Any neighbouring puffs with centroids that are within 0.96 µm of the first centroid are assigned to this site. The centre of the site is then redefined as the centre of the region enclosing all associated puffs. The process is then repeated to identify any additional puffs that may now reside within 0.96 µm of the new centre of the site. All puffs falling within the site are excluded from further consideration. The process of site identification is then repeated with the next ranked puff, and the site identification process continues until every Ca^2+^ puff has been assigned to a site. The final output from *SiteMapper* is a list of all Ca^2+^ release sites and their coordinates, which can then be used to analyse their distribution and the frequency with which each evokes Ca^2+^ puffs. *SiteMapper* was validated by overlaying the elliptical region defining each site with the centroids of the enclosed puffs (Fig. S2), and then visually confirming the number of enclosed puffs and the location of each site centre with the output from *SiteMapper*.

### Statistical analysis

Results are presented as means±s.e.m. unless indicated otherwise. Statistical comparisons used ANOVA and Student's *t*-tests, with the Bonferroni correction used for multiple comparisons. Significance is denoted by: *P<0.05, **P<0.01, ***P<0.001 and ****P<0.0001.

## Supplementary Material

Supplementary information

## References

[JCS208520C1] Alzayady K. J., Wang L., Chandrasekhar R., Wagner L. E. II, Van Petegem F. and Yule D. I. (2016). Defining the stoichiometry of inositol 1,4,5-trisphosphate binding required to initiate Ca^2+^ release. *Sci. Signal.* 9, ra35 10.1126/scisignal.aad6281 27048566 PMC4850551

[JCS208520C2] Bootman M. D., Berridge M. J. and Lipp P. (1997a). Cooking with calcium: the recipes for composing global signals from elementary events. *Cell* 91, 367-373. 10.1016/S0092-8674(00)80420-1 9363945

[JCS208520C3] Bootman M., Niggli E., Berridge M. and Lipp P. (1997b). Imaging the hierarchical Ca^2+^ signalling system in HeLa cells. *J. Physiol.* 499, 307-314. 10.1113/jphysiol.1997.sp021928 9080361 PMC1159306

[JCS208520C4] Chalmers M., Schell M. J. and Thorn P. (2006). Agonist-evoked inositol trisphosphate receptor (IP_3_R) clustering is not dependent on changes in the structure of the endoplasmic reticulum. *Biochem. J.* 394, 57-66. 10.1042/BJ20051130 16274363 PMC1386003

[JCS208520C5] Charles A. C., Merrill J. E., Dirksen E. R. and Sanderson M. J. (1991). Intercellular signaling in glial cells: calcium waves and oscillations in response to mechanical stimulation and glutamate. *Neuron* 6, 983-992. 10.1016/0896-6273(91)90238-U 1675864

[JCS208520C6] Dakin K. and Li W.-H. (2007). Cell membrane permeable esters of d-*myo*-inositol 1,4,5-trisphosphate. *Cell Calcium* 42, 291-301. 10.1016/j.ceca.2006.12.003 17307252

[JCS208520C7] Delmas P., Wanaverbecq N., Abogadie F. C., Mistry M. and Brown D. A. (2002). Signaling microdomains define the specificity of receptor-mediated InsP_3_ pathways in neurons. *Neuron* 34, 209-220. 10.1016/S0896-6273(02)00641-4 11970863

[JCS208520C8] Dickinson G. D. and Parker I. (2013). Factors determining the recruitment of inositol trisphosphate receptor channels during calcium puffs. *Biophys. J.* 105, 2474-2484. 10.1016/j.bpj.2013.10.028 24314078 PMC3853323

[JCS208520C9] Dickinson G. D., Ellefsen K. L., Dawson S. P., Pearson J. E. and Parker I. (2016). Hindered cytoplasmic diffusion of inositol trisphosphate restricts its cellular range of action. *Sci. Signal.* 9, ra108 10.1126/scisignal.aag1625 27919026 PMC5516629

[JCS208520C10] Ellefsen K. L., Settle B., Parker I. and Smith I. F. (2014). An algorithm for automated detection, localization and measurement of local calcium signals from camera-based imaging. *Cell Calcium* 56, 147-156. 10.1016/j.ceca.2014.06.003 25047761 PMC4162823

[JCS208520C11] Foskett J. K., White C., Cheung K.-H. and Mak D.-O. D. (2007). Inositol trisphosphate receptor Ca^2+^ release channels. *Physiol. Rev.* 87, 593-658. 10.1152/physrev.00035.2006 17429043 PMC2901638

[JCS208520C12] Geyer M., Huang F., Sun Y., Vogel S. M., Malik A. B., Taylor C. W. and Komarova Y. A. (2015). Microtubule-associated protein EB3 regulates IP_3_ receptor clustering and Ca^2+^ signaling in endothelial cells. *Cell Rep.* 12, 79-89. 10.1016/j.celrep.2015.06.001 26119739 PMC4487770

[JCS208520C13] Kasai H., Li Y. X. and Miyashita Y. (1993). Subcellular distribution of Ca^2+^ release channels underlying Ca^2+^ waves and oscillations in exocrine pancreas. *Cell* 74, 669-677. 10.1016/0092-8674(93)90514-Q 8395348

[JCS208520C14] Lock J. T., Smith I. F. and Parker I. (2017). Comparison of Ca^2+^ puffs evoked by extracellular agonists and photoreleased IP_3_. *Cell Calcium* 63, 43-47. 10.1016/j.ceca.2016.11.006 28108028 PMC5459673

[JCS208520C15] López-Sanjurjo C. I., Tovey S. C., Prole D. L. and Taylor C. W. (2013). Lysosomes shape Ins(1,4,5)*P*_3_-evoked Ca^2+^ signals by selectively sequestering Ca^2+^ released from the endoplasmic reticulum. *J. Cell Sci.* 126, 289-300. 10.1242/jcs.116103 23097044 PMC3603520

[JCS208520C16] Marchant J. S. and Parker I. (2001). Role of elementary Ca^2+^ puffs in generating repetitive Ca^2+^ oscillations. *EMBO J.* 20, 65-76. 10.1093/emboj/20.1.65 11226156 PMC140189

[JCS208520C17] Marchant J., Callamaras N. and Parker I. (1999). Initiation of IP_3_-mediated Ca^2+^ waves in *Xenopus* oocytes. *EMBO J.* 18, 5285-5299. 10.1093/emboj/18.19.5285 10508162 PMC1171599

[JCS208520C18] Nakamura H., Bannai H., Inoue T., Michikawa T., Sano M. and Mikoshiba K. (2012). Cooperative and stochastic calcium releases from multiple calcium puff sites generate calcium microdomains in intact Hela cells. *J. Biol. Chem.* 287, 24563-24572. 10.1074/jbc.M111.311399 22637479 PMC3397881

[JCS208520C19] Olson M. L., Sandison M. E., Chalmers S. and McCarron J. G. (2012). Microdomains of muscarinic acetylcholine and Ins(1,4,5)*P*_3_ receptors create ‘Ins(1,4,5)*P*_3_ junctions’ and sites of Ca^2+^ wave initiation in smooth muscle. *J. Cell Sci.* 125, 5315-5328. 10.1242/jcs.105163 22946060 PMC3561854

[JCS208520C20] Parker I. and Smith I. F. (2010). Recording single-channel activity of inositol trisphosphate receptors in intact cells with a microscope, not a patch clamp. *J. Gen. Physiol.* 136, 119-127. 10.1085/jgp.200910390 20660654 PMC2912063

[JCS208520C21] Parker I., Choi J. and Yao Y. (1996). Elementary events of InsP_3_-induced Ca^2+^ liberation in *Xenopus* oocytes: hot spots, puffs and blips. *Cell Calcium* 20, 105-121. 10.1016/S0143-4160(96)90100-1 8889202

[JCS208520C22] Prole D. L. and Taylor C. W. (2016). Inositol 1,4,5-trisphosphate receptors and their protein partners as signalling hubs. *J. Physiol.* 594, 2849-2866. 10.1113/JP271139 26830355 PMC4887697

[JCS208520C23] Rahman T. U., Skupin A., Falcke M. and Taylor C. W. (2009). Clustering of IP_3_ receptors by IP_3_ retunes their regulation by IP_3_ and Ca^2+^. *Nature* 458, 655-659. 10.1038/nature07763 19348050 PMC2702691

[JCS208520C24] Rooney T. A., Sass E. J. and Thomas A. P. (1990). Agonist-induced cytosolic calcium oscillations originate from a specific locus in single hepatocytes. *J. Biol. Chem.* 265, 10792-10796. 2113061

[JCS208520C25] Short A. D., Winston G. P. and Taylor C. W. (2000). Different receptors use inositol trisphosphate to mobilize Ca^2+^ from different intracellular pools. *Biochem. J.* 351, 683-686. 10.1042/bj3510683 11042123 PMC1221408

[JCS208520C26] Simpson P. B., Mehotra S., Lange G. D. and Russell J. T. (1997). High density distribution of endoplasmic reticulum proteins and mitochondria at specialized Ca^2+^ release sites in oligodendrocyte processes. *J. Biol. Chem.* 272, 22654-22661. 10.1074/jbc.272.36.22654 9278423

[JCS208520C27] Smith I. F. and Parker I. (2009). Imaging the quantal substructure of single IP_3_R channel activity during Ca^2+^ puffs in intact mammalian cells. *Proc. Natl. Acad. Sci. USA* 106, 6404-6409. 10.1073/pnas.0810799106 19332787 PMC2669345

[JCS208520C28] Smith I. F., Wiltgen S. M. and Parker I. (2009a). Localization of puff sites adjacent to the plasma membrane: functional and spatial characterization of Ca^2+^ signaling in SH-SY5Y cells utilizing membrane-permeant caged IP_3_. *Cell Calcium* 45, 65-76. 10.1016/j.ceca.2008.06.001 18639334 PMC2666303

[JCS208520C29] Smith I. F., Wiltgen S. M., Shuai J. and Parker I. (2009b). Ca^2+^ puffs originate from preestablished stable clusters of inositol trisphosphate receptors. *Sci. Signal.* 2, ra77 10.1126/scisignal.2000466 19934435 PMC2897231

[JCS208520C30] Steele E. M. and Steele D. S. (2014). Automated detection and analysis of Ca^2+^ sparks in x-y image stacks using a thresholding algorithm implemented within the open-source image analysis platform ImageJ. *Biophys. J.* 106, 566-576. 10.1016/j.bpj.2013.12.040 24507597 PMC3944640

[JCS208520C31] Taylor C. W. and Konieczny V. (2016). IP_3_ receptors: take four IP_3_ to open. *Sci. Signal.* 9, pe1 10.1126/scisignal.aaf6029 27048564 PMC5019202

[JCS208520C32] Thillaiappan N. B. (2017). Dynamics and functional architecture of native inositol 1,4,5-trisphosphate receptors. *PhD thesis*, University of Cambridge, Cambridge, UK.

[JCS208520C33] Thomas D., Lipp P., Tovey S. C., Berridge M. J., Li W., Tsien R. Y. and Bootman M. D. (2000). Microscopic properties of elementary Ca^2+^ release sites in non-excitable cells. *Curr. Biol.* 10, 8-15. 10.1016/S0960-9822(99)00258-4 10660296

[JCS208520C34] Thurley K., Tovey S. C., Moenke G., Prince V. L., Meena A., Thomas A. P., Skupin A., Taylor C. W. and Falcke M. (2014). Reliable encoding of stimulus intensities within random sequences of intracellular Ca^2+^ spikes. *Sci. Signal.* 7, ra59 10.1126/scisignal.2005237 24962706 PMC4092318

[JCS208520C35] Tong J., Du G. G., Chen S. R. W. and MacLennan D. H. (1999). Hek-293 cells possess a carbachol- and thapsigargin-sensitive intracellular Ca^2+^ store that is responsive to stop-flow medium changes and insensitive to caffeine and ryanodine. *Biochem. J.* 343, 39-44. 10.1042/bj3430039 10493909 PMC1220521

[JCS208520C36] Tovey S. C. and Taylor C. W. (2013). Cyclic AMP directs inositol (1,4,5)-trisphosphate-evoked Ca^2+^ signalling to different intracellular Ca^2+^ stores. *J. Cell Sci.* 126, 2305-2313. 10.1242/jcs.126144 23525004 PMC3672942

[JCS208520C37] Tovey S. C., De Smet P., Lipp P., Thomas D., Young K. W., Missiaen L., De Smedt H., Parys J., Berridge M. J., Thuring J. et al. (2001). Calcium puffs are generic InsP_3_-activated elementary calcium signals and are downregulated by prolonged hormonal stimulation to inhibit cellular calcium responses. *J. Cell Sci.* 114, 3979-3989. 11739630 10.1242/jcs.114.22.3979

[JCS208520C38] Tovey S. C., Dedos S. G., Taylor E. J. A., Church J. E. and Taylor C. W. (2008). Selective coupling of type 6 adenylyl cyclase with type 2 IP_3_ receptors mediates direct sensitization of IP_3_ receptors by cAMP. *J. Cell Biol.* 183, 297-311. 10.1083/jcb.200803172 18936250 PMC2568025

[JCS208520C39] Tu J. C., Xiao B., Yuan J. P., Lanahan A. A., Leoffert K., Li M., Linden D. J. and Worley P. F. (1998). Homer binds a novel proline-rich motif and links group 1 metabotropic glutamate receptors with IP_3_ receptors. *Neuron* 21, 717-726. 10.1016/S0896-6273(00)80589-9 9808459

[JCS208520C40] Wiltgen S. M., Dickinson G. D., Swaminathan D. and Parker I. (2014). Termination of calcium puffs and coupled closings of inositol trisphosphate receptor channels. *Cell Calcium* 56, 157-168. 10.1016/j.ceca.2014.06.005 25016315 PMC4162808

[JCS208520C41] Zhao H., Khademazad M. and Muallem S. (1990). Agonist-mediated Ca^2+^ release in permeabilized UMR-106-01 cells. Transport properties and generation of inositol 1,4,5-trisphosphate. *J. Biol. Chem.* 265, 14822-14827. 2203762

