# Supplementary material for: Endogenous signalling pathways and caged IP3 evoke Ca2+ puffs at the same abundant immobile intracellular sites
Source: J Cell Sci. 2017 Nov 1;130(21):3728–39. doi: 10.1242/jcs.208520 (PMC5702060; doi:10.1242/jcs.208520)
Supplement: Supplementary information [file joces-130-208520-s1.pdf]

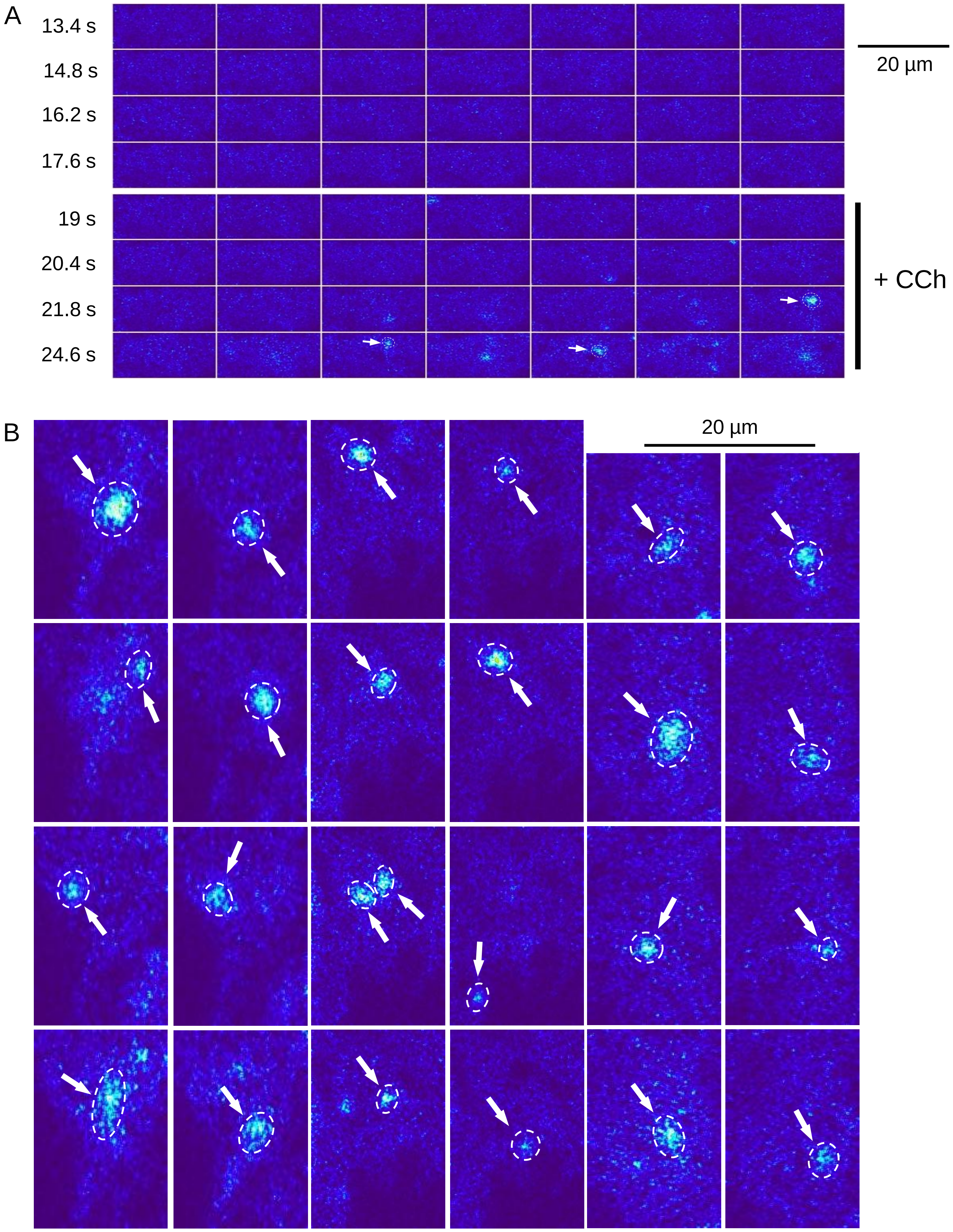

Fig. S1. Further examples of Ca^{2+} puffs evoked by carbachol in HEK293 cells. (A) TIRFM images show ΔF in pseudocolour for a HEK293 cell before and after stimulation with carbachol (10 µM at 19 s). The layout is the same as shown in Figure 2A, with every 5th frame shown within the indicated time intervals. Ca^{2+} puffs detected by *PuffFinder* are indicated by white arrows. (B) Typical examples of Ca^{2+} puffs (arrows) evoked by carbachol and detected by *PuffFinder* are shown on an enlarged scale.

Journal of Cell Science • Supplementary information

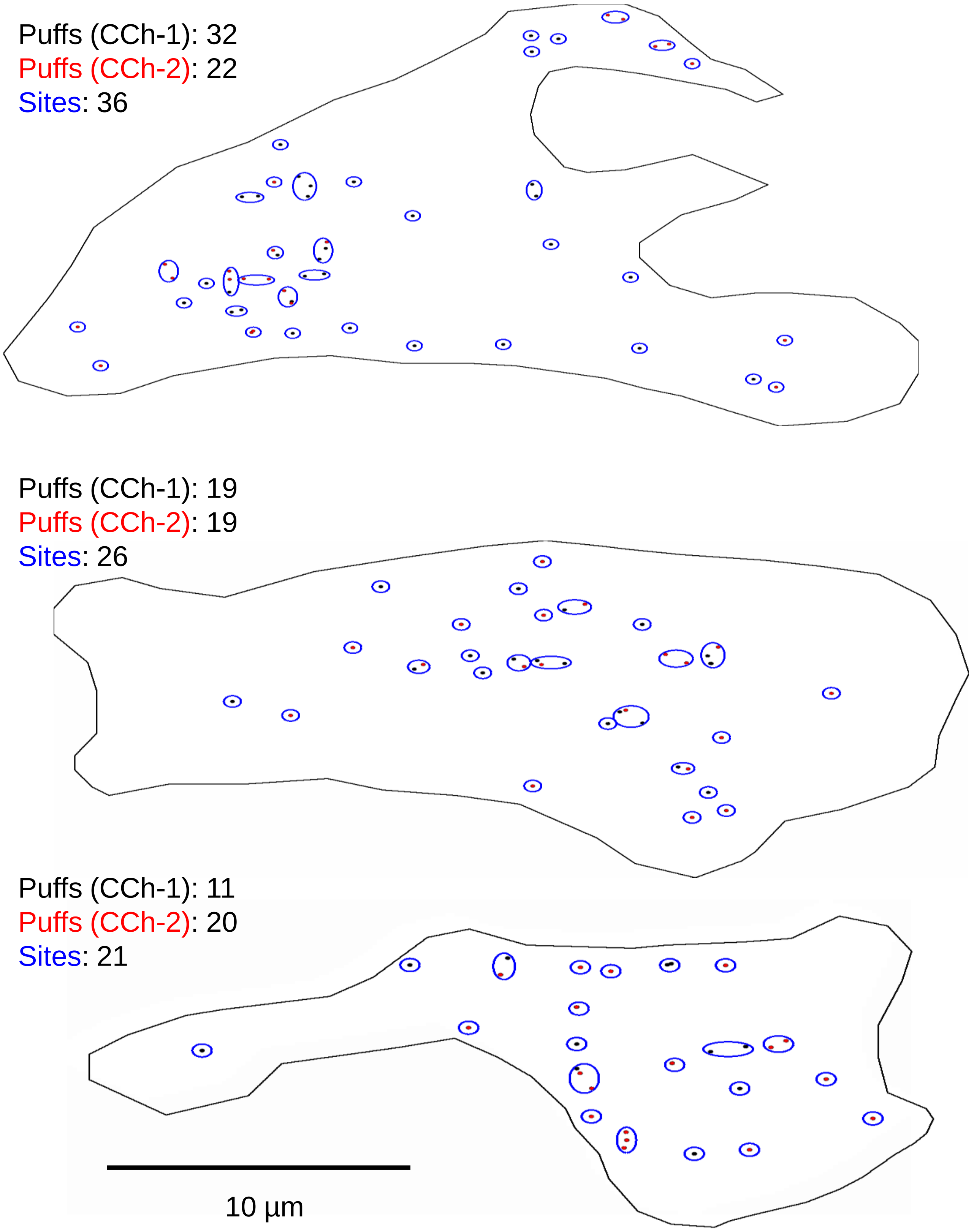

Fig. S2. Use of *SiteMapper* to attribute Ca^{2+} puffs to Ca^{2+} release sites. Three representative HEK293 cells show the centroids of all Ca^{2+} puffs detected by *PuffFinder* during the first (black) and second (red) stimulation with carbachol (10 µM) (see Fig. 3A for the stimulation protocol). *SiteMapper* was then used to identify the Ca^{2+} release sites (blue circles). The numbers of Ca^{2+} puffs detected during each stimulus and the sites identified are shown for each cell.

Journal of Cell Science • Supplementary information

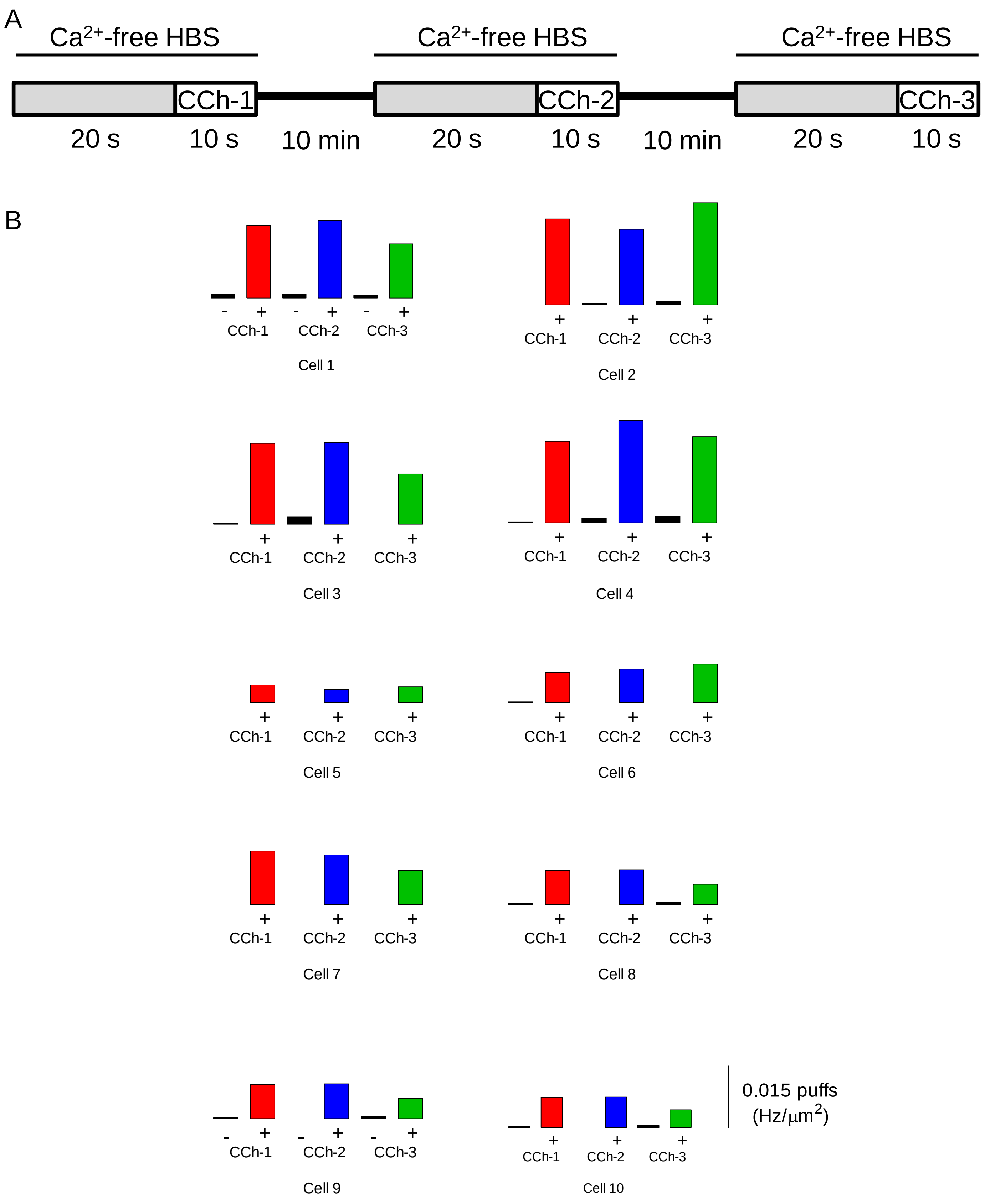

Fig. S3. Responses of individual HEK293 cells to repeated stimulation with carbachol. (A) The stimulation protocol shown was used to stimulate HEK293 cells with three sequential challenges with carbachol (CCh, 10 μM) interspersed with periods (10 min) of recovery.
(B) Results, for 10 individual cells show the density of the Ca^{2+} puffs detected during each pre-incubation period and subsequent stimulation with CCh. Summary results are shown in Figure 3B.

Journal of Cell Science • Supplementary information

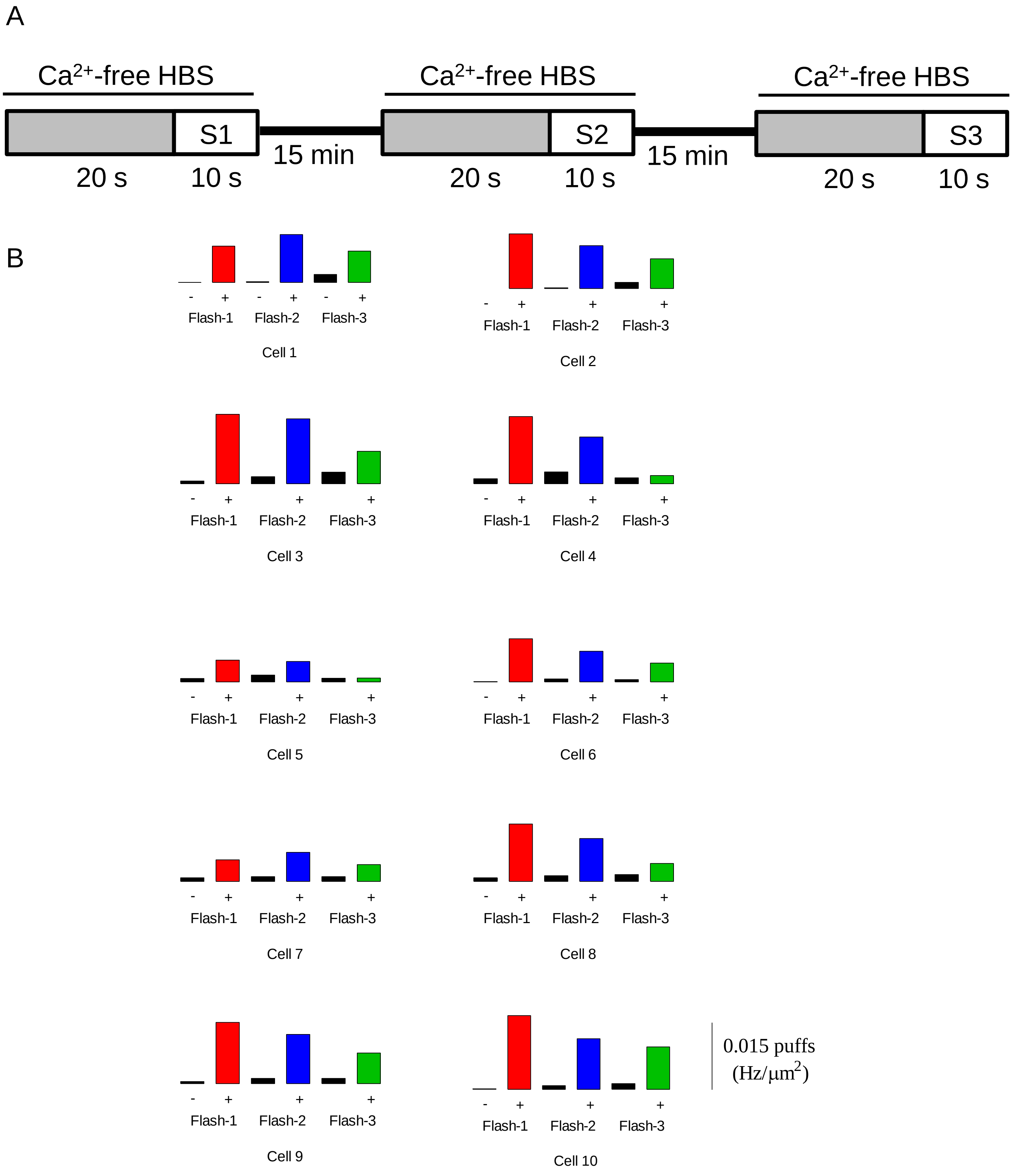

Fig. S4. Responses of individual HEK293 cells to repeated photolysis of caged IP_3. (A) The stimulation protocol shown was used to stimulate HEK293 cells with three sequential challenges with UV light (S1-S3) to photolyse caged IP_3 interspersed with periods (15 min) of recovery. (B) Results, for 10 individual cells show the density of the Ca^{2+} puffs detected during each pre-incubation period and after each photorelease of IP_3.

Journal of Cell Science • Supplementary information

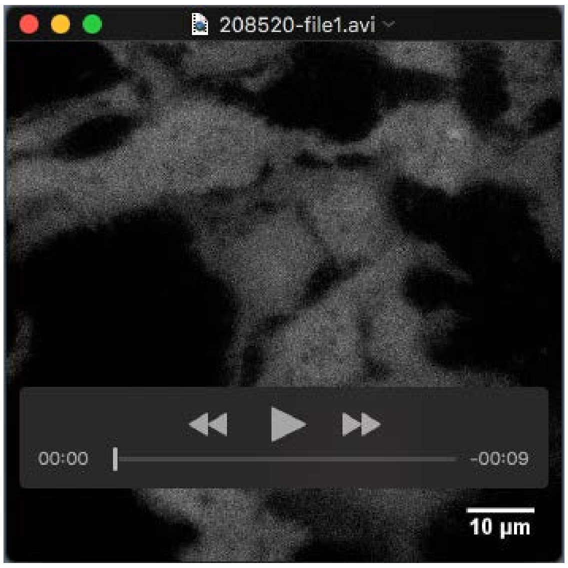

Video 1. Typical TIRFM recording of Ca^{2+} puffs from a field of HEK293 cell stimulated with carbachol (10 µM) after ~20 s. The video, showing unprocessed images, was captured and replayed at 25 frames/s (fps). Scale bar = 10 µm.

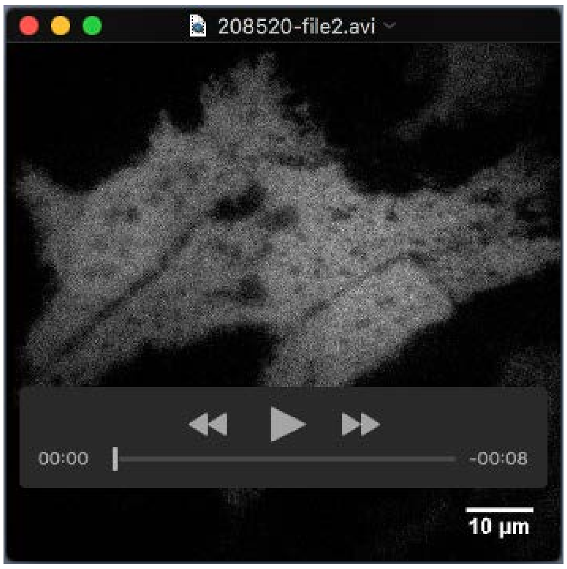

Video 2. Typical TIRFM recording of Ca^{2+} puffs from a field of HEK293 cell loaded with ci-IP_3 and stimulated with a UV-flash (40 ms) after 20 s. The video, showing unprocessed images, was captured and replayed at 25 fps. Scale bar = 10 µm.

Journal of Cell Science • Supplementary information

Table S1. Effects of three sequential challenges with carbachol on Ca^{2+} puffs and detection of initiation sites.

Figure 3A shows the protocol used to stimulate HEK293 cells three times with carbachol (CCh, 10 µM). The responses evoked during the last 2 s after each CCh addition were analysed (see text for details). Results (from 16 cells) show the number of puffs detected during each stimulation period, the number of active sites, and the number of new sites identified during each successive stimulation (summarised in Figure 3D). From the numbers of active sites/cell (17 ± 1.8) and new active sites/cell (12 ± 1.1) detected during the second stimulation with carbachol, we calculate that only 26 ± 3% of all sites responded during this challenge (which detected 17 ± 1.8 sites). From this we estimate *N* = 65 ± 7 sites/cell.

Cell	Number of puffs			Number of active sites			New active sites		
	CCh-1	CCh-2	CCh-3	CCh-1	CCh-2	CCh-3	CCh-1	CCh-2	CCh-3
1	36	34	25	28	27	20	28	21	12
2	63	39	44	46	30	39	46	17	22
3	24	27	20	17	23	16	17	15	10
4	16	19	14	13	17	12	13	14	8
5	37	34	33	27	26	22	27	18	6
6	5	18	14	5	15	13	5	14	11
7	9	7	6	9	7	6	9	6	4
8	28	14	7	22	13	5	22	12	4
9	12	8	9	10	8	8	10	4	5
10	18	16	10	11	11	9	11	9	7
11	67	32	23	52	24	21	52	13	10
12	15	13	27	13	12	21	13	10	13
13	19	19	8	15	18	7	15	11	4
14	11	20	16	9	14	12	9	12	10
15	32	22	34	25	15	27	25	11	18
16	17	16	8	12	10	7	12	7	4
mean	26	21	19	20	17	15	20	12	9
SD	18	10	11	13	7	9	13	4	5
SEM	4.5	2.4	2.9	3.3	1.8	2.3	3.3	1.1	1.3

Journal of Cell Science • Supplementary information

Table S2. Estimation of the total number of Ca^{2+} release sites/cell from the distribution of carbachol-evoked Ca^{2+} puffs between sites.

From the results shown in Figure 3C, the first challenge with carbachol (CCh-1) was used to provide the observed number of sites responding with 1-5 puffs for each cell (no sites evoked more than 5 puffs). We then used the Poisson distribution to estimate the probability of a puff initiating at a site during the stimulation period (2 s). For a Poisson distribution: $P(\mathrm{n}) = \mathrm{e}^{-\mu}.\frac{\mu^{\mathrm{n}}}{\mathrm{n}!}$ where P(n) is the probability of observing n puffs at a site, and μ is the mean number of puffs/site. Hence, $P(1) = \mathrm{Obs1}/N = \mathrm{e}^{-\mu}.\mu$ and $P(2) = \mathrm{Obs2}/N = \mathrm{e}^{-\mu}.\frac{\mu^2}{2}$ where N is the total number of sites/cell. Since $P(1)/P(2) = \mathrm{Obs1}/\mathrm{Obs2} = 2/\mu$, we estimate μ for each cell from: $\mu = \frac{2\mathrm{Obs2}}{\mathrm{Obs1}}$; and from $\mathrm{Obs1}/N = \mathrm{e}^{-\mu}.\mu$, we estimate N for each cell: $N = \frac{\mathrm{Obs1}}{\mu \mathrm{e}^{-\mu}}$. The analysis suggests there are 70 ± 13 release sites/cell. The same analyses applied to responses to the second and third carbachol challenges provided similar estimates of N: 75 ± 11 and 74 ± 17, respectively. Exclusion of the 4 cells where no sites responded with a second Ca^{2+} puff could bias the analysis, but applying the same method to pooled data for all 16 cells provided an estimate of N = 59, which is not significantly different from that determined from analyses of individual cells. Since the method presented in Table S1 to estimate N is not prone to errors from stochastic variations in the small number of sites that evoke 2 Ca^{2+} puffs, we used it for most estimates of N.

Cell	Observed number of sites responding with 1-5 puffs n					μ	N
	1	2	3	4	5		
1	23	3	1	1	0	0.261	114
2	34	8	3	1	0	0.471	116
3	11	5	1	0	0	0.909	30
4	11	1	1	0	0	0.182	73
5	20	4	3	0	0	0.400	75
6	5	0	0	0	0		
7	9	0	0	0	0		
8	18	2	2	0	0	0.222	101
9	9	0	1	0	0		
10	6	3	2	0	0	1.000	16
11	41	8	2	1	0	0.390	155
12	12	0	1	0	0		
13	12	2	1	0	0	0.333	50
14	7	2	0	0	0	0.571	22
15	19	5	1	0	0	0.526	61
16	8	3	1	0	0	0.750	23
Mean	15.31	2.88	1.25	0.19	0.00	0.501	70
SD	10.22	2.60	0.93	0.40	0.00	0.265	45
SEM	2.55	0.65	0.23	0.10	0.00	0.076	13

Journal of Cell Science • Supplementary information

Table S3. Effects of two sequential stimulations with UV to photolyse caged IP_3 on Ca^{2+} puffs and detection of initiation sites.

Figure 5A shows the protocol used to stimulate HEK293 cells twice with i-IP_3 released from its caged precursor by UV light. Results (from 10 cells) show the number of puffs detected during each stimulation period, the number of active sites, and the number of new sites identified during each successive stimulation (summarised in Figure 5D). From the numbers of active sites/cell (13 ± 2.5) and new active sites/cell (12 ± 2.3) detected during the second stimulation with i-IP_3, we calculate that this period of stimulation, which identified 13 ± 2.5 sites, detected only 13.0 ± 4.6% of the sites, suggesting there are 100 ± 35 sites/cell.

Cell	Number of puffs			Number of active sites			New active sites	
	Flash-1	Flash-2		Flash-1	Flash-2		Flash-1	Flash-2
1	17	21		12	16		12	15
2	15	25		12	22		12	20
3	12	9		10	7		10	7
4	34	29		29	26		29	23
5	14	10		13	9		13	8
6	15	17		13	13		13	11
7	4	3		3	2		3	1
8	22	16		17	14		17	11
9	11	23		9	19		9	18
10	13	4		11	4		11	4
mean	16	16		13	13		13	12
SD	8	9		7	8		7	7
SEM	2.5	2.8		2.1	2.5		2.1	2.3

Journal of Cell Science • Supplementary information
